# Genomic and Phenotypic Evolution of Achromobacter xylosoxidans during Chronic Airway Infections of Patients with Cystic Fibrosis

**DOI:** 10.1128/mSystems.00523-21

**Published:** 2021-06-29

**Authors:** S. M. Hossein Khademi, Migle Gabrielaite, Magnus Paulsson, Mattis Knulst, Eleni Touriki, Rasmus L. Marvig, Lisa I. Påhlman

**Affiliations:** a Department of Clinical Sciences Lund, Division of Infection Medicine, Lund University, Lund, Sweden; b Center for Genomic Medicine, Rigshospitalet, Copenhagen, Denmark; c Division of Infectious Diseases, Skåne University Hospital Lund, Lund, Sweden; d Clinical Microbiology, Labmedicin Skåne, Lund, Sweden; e Wallenberg Centre for Molecular Medicine, Lund University, Lund, Sweden; Marquette University

**Keywords:** multidrug antibiotic resistance, convergent evolution, *Achromobacter xylosoxidans*, genome-wide association study, cystic fibrosis, bacterial adaptation

## Abstract

Bacterial pathogens evolve during chronic colonization of the human host by selection for pathoadaptive mutations. One of the emerging and understudied bacterial species causing chronic airway infections in patients with cystic fibrosis (CF) is Achromobacter xylosoxidans. It can establish chronic infections in patients with CF, but the genetic and phenotypic changes associated with adaptation during these infections are not completely understood. In this study, we analyzed the whole-genome sequences of 55 clinical A. xylosoxidans isolates longitudinally collected from the sputum of 6 patients with CF. Four genes encoding regulatory proteins and two intergenic regions showed convergent evolution, likely driven by positive selection for pathoadaptive mutations, across the different clones of A. xylosoxidans. Most of the evolved isolates had lower swimming motility and were resistant to multiple classes of antibiotics, while fewer of the evolved isolates had slower growth or higher biofilm production than the first isolates. Using a genome-wide association study method, we identified several putative genetic determinants of biofilm formation, motility and β-lactam resistance in this pathogen. With respect to antibiotic resistance, we discovered that a combination of mutations in pathoadaptive genes (*phoQ* and *bigR*) and two other genes encoding regulatory proteins (*spoT* and *cpxA*) were associated with increased resistance to meropenem and ceftazidime. Altogether, our results suggest that genetic changes within regulatory loci facilitate within-host adaptation of A. xylosoxidans and the emergence of adaptive phenotypes, such as antibiotic resistance or biofilm formation.

**IMPORTANCE** A thorough understanding of bacterial pathogen adaptation is essential for the treatment of chronic bacterial infections. One unique challenge in the analysis and interpretation of genomics data is identifying the functional impact of mutations accumulated in the bacterial genome during colonization in the human host. Here, we investigated the genomic and phenotypic evolution of A. xylosoxidans in chronic airway infections of patients with CF and identified several mutations associated with the phenotypic evolution of this pathogen using genome-wide associations. Identification of phenotypes under positive selection and the associated mutations can enlighten the adaptive processes of this emerging pathogen in human infections and pave the way for novel therapeutic interventions.

## INTRODUCTION

Following colonization of the human hosts, some bacterial pathogens genetically adapt to establish chronic infections. By whole-genome sequencing of longitudinally collected isolates from patients with chronic infections, genetic changes responsible for adaptive phenotypes can be discovered (1). Airway infections of patients with cystic fibrosis (CF) have often been used as a relevant model for studies of chronic bacterial infection, where the infection often causes respiratory failure and early death (2).

One of the emerging CF pathogens that have attracted increased attention over the last years is the Gram-negative opportunistic pathogen Achromobacter xylosoxidans. Chronic infection with A. xylosoxidans in patients with CF is associated with increased exacerbation frequency and more rapid decline of respiratory function than infections with Pseudomonas aeruginosa (3, 4). Depending on the definition of infection, the observation period, and the frequency of surveillance cultures, the prevalence of A. xylosoxidans in patients with CF is estimated at 3% to 18% (5–8). However, its prevalence has reportedly increased in the last 2 decades (5, 6, 9). In addition to patients with CF, this opportunistic pathogen has been found in severe blood, middle ear, urinary tract, and respiratory infections, especially in older or immunocompromised patients (10–13). Furthermore, this pathogen is often resistant to a wide range of antibiotic classes, including but not limited to aminoglycosides, cephalosporins, and monobactams (14, 15). There are also several reports of transmission of A. xylosoxidans between patients with CF (14, 16, 17).

Multiple studies have investigated the genetic changes associated with within-host evolution and adaptation of P. aeruginosa, Staphylococcus aureus, Stenotrophomonas maltophilia, Burkholderia cenocepacia, and Burkholderia multivorans in patients with CF (18–22). However, within-host evolution and genetic adaptation of A. xylosoxidans in CF airway infection remains largely unknown. In this study, we used a collection of 55 longitudinal isolates from six patients with CF to discover the genetic determinants important for A. xylosoxidans adaptation in CF. By analysis of the phenotypic changes in biofilm formation, motility, growth and antibiotic resistance, and utilization of a genome-wide association study (GWAS), we identified several putative genetic determinants of these adaptive phenotypes in A. xylosoxidans.

## RESULTS

### Clinical isolates of A. xylosoxidans infecting patients with CF.

**FIG 1:**
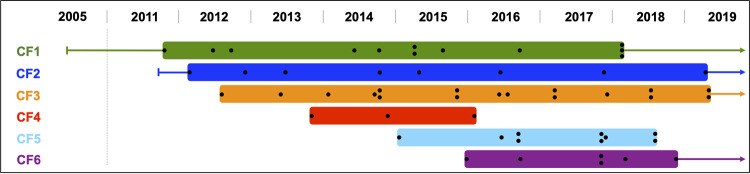
Overview of the time points when the 55 sequenced A. xylosoxidans isolates were found in the sputum of six patients with CF included in this study (Table S1B). All sequenced clinical isolates of A. xylosoxidans are represented by black dots. The colored lines show colonization of A. xylosoxidans before the first or after the last sequenced isolates. The years 2006 to 2010 are not shown, as no isolates from these years were sequenced.

A total of six patients with CF, chronically colonized by A. xylosoxidans, were identified between 2011 and 2019 at the CF center in Lund, Sweden (see Table S1A in the supplemental material). To investigate the genetic adaptation of A. xylosoxidans in the CF host airways and discover mutations responsible for altered phenotypes, we sequenced the genomes of 55 isolates collected longitudinally from these patients (median, 9 isolates per patient; range, 3 to 17) (Fig. 1). The sequenced isolates were collected over a median period of 5.0 years (range, 2.3 to 7.2). The patients’ median age was 19.9 years at first isolation of A. xylosoxidans (range, 12.1 to 36.2). For all but one patient, the first isolate included in the study was collected within 6 months from the initial detection of A. xylosoxidans in the sputum. For patient CF1, no isolate was available within the initial 6.4 years of colonization. Colonization by A. xylosoxidans continued for all but two patients (CF4 and CF5) throughout the study period. CF4 cleared A. xylosoxidans after 2.3 years, while A. xylosoxidans colonization ended in CF5 after a double lung transplant in September 2018.

10.1128/mSystems.00523-21.1 TABLE S1 Raw clinical and experimental data. Download Table S1, XLSX file, 0.1 MB. Copyright © 2021 Khademi et al. 2021 Khademi et al. https://creativecommons.org/licenses/by/4.0/ This content is distributed under the terms of the Creative Commons Attribution 4.0 International license.

### Hypermutator phenotype increases within-host genetic diversification of A. xylosoxidans.

**FIG 2:**
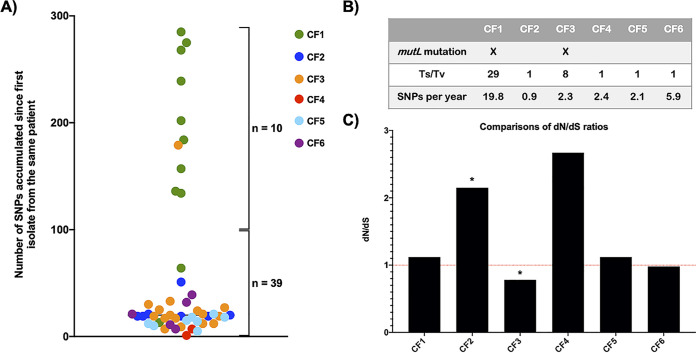
(A) Genetic distance (SNPs) between each isolate (colored circles) of the six patients and the first isolate from the same patient (Table S3). (B) Summary data from each clone on the presence of deleterious mutations in DNA MMR gene *mutL* leading to the hypermutator phenotype, the fold change of Ts/Tv (transition/transversion), and the mean substitution rate estimated with BEAST. (C) Nonsynonymous-to-synonymous mutation ratio (*dN*/*dS*) for each clone. The red dotted line at a *dN*/*dS* of 1 represents neutral evolution. The asterisk indicates significance of change in number of N over S mutations in each clone compared to those found in the entire data set excluding that clone (Fisher’s exact test, *P* < 0.05).

Initial analysis of multilocus sequence typing (MLST) using *de novo* assembled genomes showed that each of the 6 patients were infected by an individual unique clone throughout the study period (Table S1C). We therefore assigned the same nomenclature (CF1 to CF6) for clones from each patient. To gain insight into A. xylosoxidans within-host evolution in CF airways, we identified genetic variants that differentiated isolates of the same clone; i.e., we identified mutations that had accumulated during the period of infection in each of the six clones. In total, we identified 1,499 variants (median, 76; range, 12 to 931), including 837 nonsynonymous (NS) single-nucleotide polymorphism (SNPs), 165 insertions/deletions (indels), 124 intergenic SNPs, 110 intergenic indels, and 263 synonymous (S) SNPs (Table S1D). Ten of the 55 isolates had a large genetic distance (>100 SNPs) from the initial isolate of the same patient. One of the 10 isolates belonged to CF3, and the other nine isolates all belonged to CF1 (Fig. 2A). The CF3-S12a isolate, found in the latest sputum sample from this patient, contained a nonsense mutation in the DNA mismatch repair system (MMR) gene *mutL*, which was absent in all other isolates of this clone and which could explain the elevated mutation number of this isolate. Moreover, a manual inspection of the MMR system genes in the 12 isolates of CF1 showed that all nine isolates with higher genetic distance had a 112-bp deletion in *mutL*. The deletion was found in all of the following genomes from the third isolate, but the fifth isolate, which also had the deletion, was below the threshold of large genetic distance (64 SNPs relative to the first isolate) (Fig. 2A). As this deletion leads to a functional defect in MutL, it is likely responsible for the increased number of mutations in this clone. We also found higher levels of transitions to transversion (Ts/Tv) among SNPs of CF1 (Ts/Tv = 29) and CF3 (Ts/Tv = 8) than among SNPs of other clones (Ts/Tv ≈ 1) (Fig. 2B), which is in agreement with the expected effect of the MutL defects (23).

10.1128/mSystems.00523-21.3 TABLE S3 SNP distance between isolates of each clone. Download Table S3, XLSX file, 0.02 MB. Copyright © 2021 Khademi et al. 2021 Khademi et al. https://creativecommons.org/licenses/by/4.0/ This content is distributed under the terms of the Creative Commons Attribution 4.0 International license.

In the BEAST analysis of the nucleotide substitution rate of A. xylosoxidans clones infecting each patient, we found a higher estimated mean substitution rate of 19.8 SNPs/year for isolates of CF1 relative to the other clones (range, 0.9 to 5.9 SNPs/year) (Table S1E). The higher substitution rate for CF1 was consistent with the hypermutator phenotype. In contrast, the estimated mean substitution rate for isolates of CF2 was 0.9 SNPs/year, which was lower than that of all other clones. The overall average substitution rate for all clones excluding the hypermutator CF1 was 2.7 SNPs/year (Fig. 2B), which is close to the previously reported 1.9 SNPs/year in CF adapted isolates of A. xylosoxidans genomes (24). In comparison, the mean substitution rates of CF adapted genomes of P. aeruginosa and B. multivorans were reported at 2.6 and 2.4 SNPs/year, respectively (22, 25).

### A. xylosoxidans clones have varying evolutionary dynamics.

To identify evidence for either positive or negative selection of mutations in isolates of each clone, we calculated the relative rates of NS and S substitutions (*dN*/*dS*) (Fig. 2C). While mutations in isolates of CF1, CF5, and CF6 occurred mostly under neutral selection, *dN*/*dS* was higher for CF2 and CF4. However, we found a significant increase of *dN*/*dS* only in CF2 compared to other clones (Fisher’s exact test, *P =* 0.04), possibly explainable by the lower total number of substitutions in CF4. As isolates of CF2 also had a lower substitution rate, it is clear that the majority of substitutions in this clone are NS. In contrast, isolates of CF3 exhibit a sign of negative selection with a *dN*/*dS* of 0.8, and the *dN*/*dS* ratio is significantly lower in this clone than the other clones (Fisher’s exact test, *P =* 0.01). Interestingly, in-depth analysis of the selection signature in CF3 showed two significantly different phases of selection for the earlier eight isolates (*dN*/*dS* = 0.5) compared to the later nine isolates (*dN*/*dS* = 1.1) (Fig. S1) (Fisher’s exact test, *P =* 0.01). However, one major caveat of this evolutionary dynamics analysis is that the calculated ratio of substitution rates (*dN*/*dS*) was performed for clones from a single population; thus, the strength of positive selection is likely underestimated by the presence of polymorphisms that are not yet fixed in the population (26).

10.1128/mSystems.00523-21.6 FIG S1 RAxML phylogenetic trees showing the SNP relatedness between isolates of the six clones. The occurrence point of each pathoadaptive mutation is shown on the trees. For CF3, two different phases of selection are highlighted in the tree and the number of S or NS SNPs in each phase is shown. Note that no phylogenetic tree was made for CF4, since there were only 3 isolates from this clone. Download FIG S1, PDF file, 0.1 MB. Copyright © 2021 Khademi et al. 2021 Khademi et al. https://creativecommons.org/licenses/by/4.0/ This content is distributed under the terms of the Creative Commons Attribution 4.0 International license.

### Independent clones of A. xylosoxidans show convergent evolution in pathoadaptive loci.

**FIG 3:**
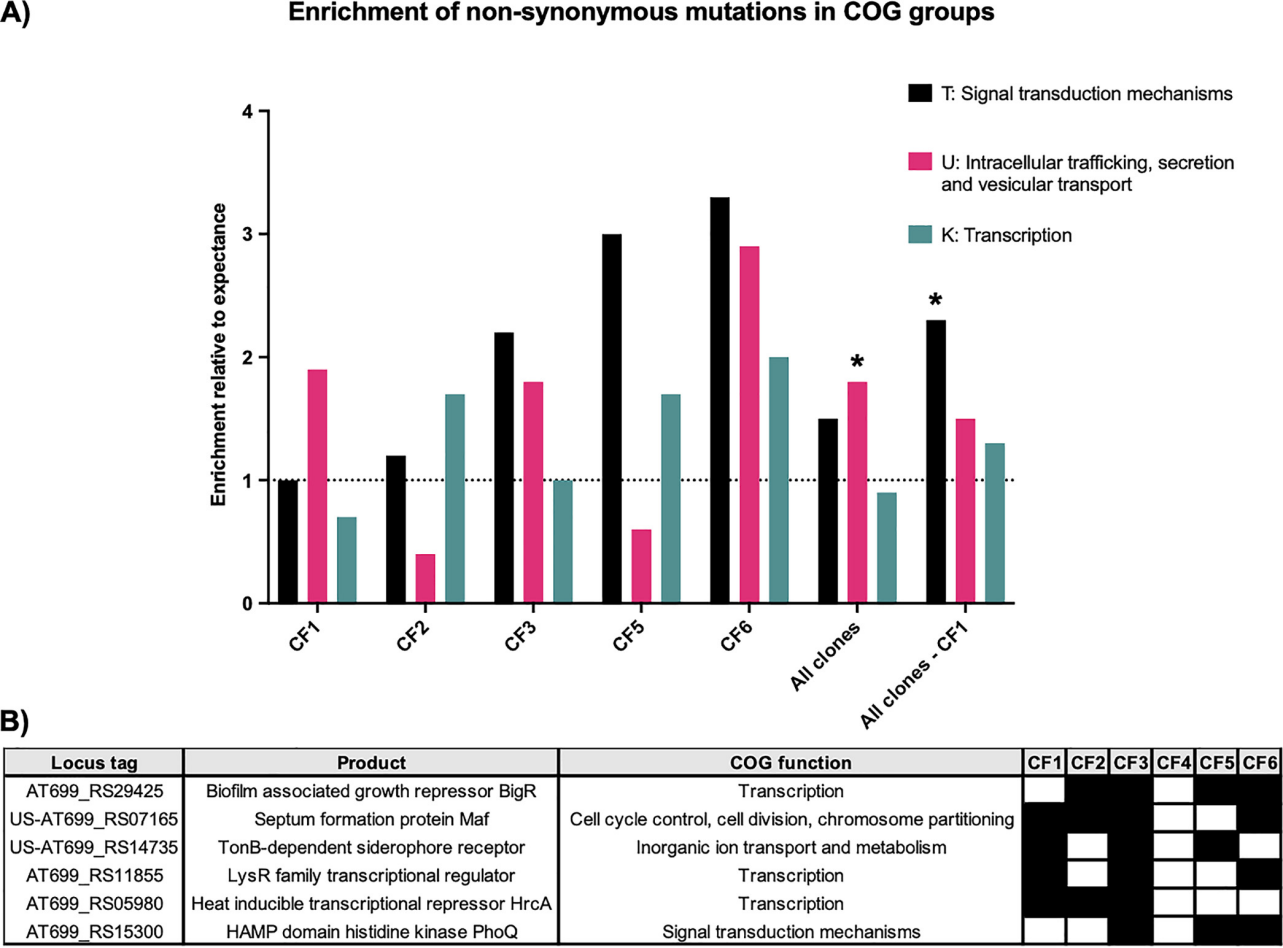
(A) Enrichment of genes from three clusters of orthologous groups (COGs) of proteins among NS mutations compared to the normal presence of each COG in the A. xylosoxidans genome. The analysis included NS mutations from all clones, all clones except CF1, and for each of the five clones (excluding CF4, which had too few NS mutations for the analysis). The asterisk indicates significance of COG genes presence versus absence in the data set compared to the genome (Fisher’s exact test, Bonferroni adjustment, *P* < 0.05). (B) The six frequently mutated (pathoadaptive) loci across isolates of the six clones (Table S1G). The presence of mutation in isolates of each clone is shown by the black square. US, upstream of intergenic loci.

To explore the function of genes enriched for mutations in CF-adapted A. xylosoxidans, we analyzed the distribution of NS mutations within 21 different clusters of orthologous groups (COG) of proteins found in A. xylosoxidans genomes (Table S1F). When NS mutations from all clones were analyzed (Fig. 3A), we found a significant enrichment of the group “Intracellular trafficking, secretion and vesicular transport” (U) among NS mutations compared with the presence of this group in the reference genome (Fisher’s exact test, *P =* 0.001). Surprisingly, COG U was overrepresented by the contribution of CF1, since when NS mutations of this clone were excluded from the analysis, we found a significant enrichment of the group “Signal transduction mechanisms” (T) (Fisher’s exact test, *P =* 0.001). We also observed some divergence in other groups targeted by NS mutations from each clone individually. For example, the group “Transcription” (K) was enriched in targets of CF2, CF5, and CF6 while being unchanged or even depleted in those of CF3 and CF1.

Despite these divergences in NS mutational targets, we still found consistent patterns of convergent evolution in certain genetic loci across independent clones. Using an intuitive approach based on length of the loci and the expected mutation number with respect to the normal distribution of mutations across the genome, we found four genes and two intergenic regions to be significantly enriched by parallel independent mutations across different clones (Fig. 3B). As these six loci are likely to be involved in pathogen host adaptation, we refer to them as pathoadaptive loci. All four genes were mutated only by NS mutations. The most frequently mutated gene across isolates of four clones was a transcriptional regulator gene with a product putatively identified as the biofilm-associated growth repressor BigR. As suggested by the annotation, this protein has been associated with regulation of biofilm growth in response to hydrogen sulfite in Xylella fastidiosa and Acinetobacter baumannii (27, 28). Two of the other pathoadaptive genes, encoding a LysR-family transcriptional regulator and the heat-inducible transcriptional repressor HrcA, were also transcriptional regulator genes. The fourth pathoadaptive gene encoded a putative HAMP domain histidine kinase PhoQ, which is associated with virulence, motility, and polymyxin resistance in Salmonella enterica serovar Typhimurium and P. aeruginosa (29–31). With the exception of *hrcA*, all other genes were found to be pathoadaptive in CF-adapted A. xylosoxidans from another study, confirming the relevance of these loci in genetic adaptation of A. xylosoxidans in CF (24). Moreover, the two intergenic loci had mutations upstream of genes encoding the septum formation protein Maf and a TonB siderophore receptor, which may be involved in metal uptake through siderophores similar to metal uptake in P. aeruginosa (32, 33).

### Evolution of adaptive phenotypes in independent clones of A. xylosoxidans.

To understand the phenotypic evolution of A. xylosoxidans in CF, we tested the 55 sequenced isolates with some of the known adaptive phenotypes from other bacterial species found in the CF model (22, 34).

**FIG 4:**
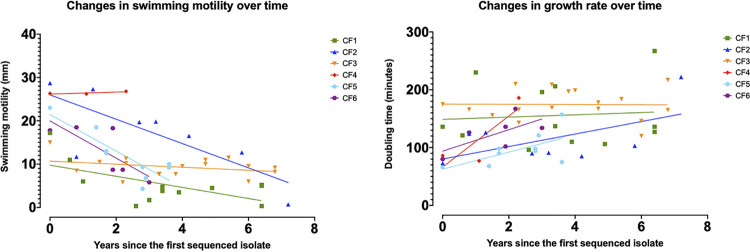
Mean swimming motility and doubling times for each of the 55 clinical isolates of A. xylosoxidans (Table S1H and I). The values are plotted against the number of years the isolate colonized the patient since the first isolate (Table S1B) to follow the change of the phenotype over colonization time in CF. The colored lines are the best-fit linear regression for isolates of each clone (colored symbols).

In our study, the analysis of swimming motility showed that this phenotype is gradually lost over time following the initial colonization (Fig. 4). While evolved isolates of CF2, CF5, and CF6 had a rapid decline in motility over longer colonization time, decrease in motility was less pronounced for evolved isolates of CF1 and CF3. This may be because the first isolates of CF1 and CF3 already had lower swimming motility than first isolates from other patients. Moreover, unlike other clones, all three isolates of CF4 had an unchanged swimming motility. Looking at the possible mutations for the observed phenotype (Table S2), we found an indel in the gene encoding the flagellar protein FliS in isolate CF1-S4 that may be responsible for the diminished swimming motility of this isolate. We also identified NS mutations in the two genes encoding FimV and a fimbrial usher protein (AT699_RS04965) in isolate CF1-S9c, which had no swimming motility (Fig. S2).

10.1128/mSystems.00523-21.2 TABLE S2 SNP and indel mutations that have accumulated between A. xylosoxidans isolates of the same clone during colonization in each patient. Download Table S2, XLSX file, 0.3 MB. Copyright © 2021 Khademi et al. 2021 Khademi et al. https://creativecommons.org/licenses/by/4.0/ This content is distributed under the terms of the Creative Commons Attribution 4.0 International license.

10.1128/mSystems.00523-21.7 FIG S2 Doubling times of the isolates during exponential growth in the minimal ABTGC medium. The asterisk denotes a significantly changed doubling time of an isolate from the first isolate of the same patient (Tukey’s HSD multiple comparison, *P* < 0.05). The raw data are available in Table S1I. Download FIG S2, PDF file, 0.04 MB. Copyright © 2021 Khademi et al. 2021 Khademi et al. https://creativecommons.org/licenses/by/4.0/ This content is distributed under the terms of the Creative Commons Attribution 4.0 International license.

Previous studies have shown that P. aeruginosa isolates converge toward a lower growth rate following adaptation in the CF lung (34). We also found some of the evolved isolates of A. xylosoxidans from CF2, CF4, CF5, and CF6 to be slower growing in the ABT minimal medium with glucose and Casamino Acids (Fig. 4). In contrast, most evolved isolates of CF1 and CF3 were relatively unchanged over time with respect to growth in minimal medium; likely because the first isolates of these two clones already had a lower growth rate than isolates of the other clones.

Previous studies reported that most A. xylosoxidans strains isolated from patients with CF can make biofilms *in vitro* (6). In contrast, we found that a total of only 15 isolates from CF1 (*n* = 7), CF2 (*n* = 1), CF4 (*n* = 1), CF5 (*n* = 4), and CF6 (*n* = 2) could form biofilms (optical density at 550 nm [OD_550_] > 0.3). Furthermore, while some of the evolved isolates of CF2, CF4, CF5, and CF6 had increased biofilm formation, most of the evolved CF1 isolates had decreased biofilm formation (Fig. S4).

10.1128/mSystems.00523-21.8 FIG S3 Swimming motility of the isolates. The raw data are available in Table S1H. Download FIG S3, PDF file, 0.04 MB. Copyright © 2021 Khademi et al. 2021 Khademi et al. https://creativecommons.org/licenses/by/4.0/ This content is distributed under the terms of the Creative Commons Attribution 4.0 International license.

10.1128/mSystems.00523-21.9 FIG S4 Biofilm formation of the isolates. The raw data are available in Table S1J. Download FIG S4, PDF file, 0.04 MB. Copyright © 2021 Khademi et al. 2021 Khademi et al. https://creativecommons.org/licenses/by/4.0/ This content is distributed under the terms of the Creative Commons Attribution 4.0 International license.

To dissect the genetic background behind development of these three phenotypes in A. xylosoxidans, we used a k-mer-based GWAS method (35). Utilizing this method, we identified three genetic components as being significantly associated with the motility and 29 components with the biofilm phenotypes (*q* < 0.05) (Table S1K to L). Some of the identified components were in genes with putative homologs that have previously been linked to these phenotypes in other species of bacteria, demonstrating their probable relevance for the observed phenotypes of A. xylosoxidans colonizing the CF host (Table 1).

**TABLE 1:** Genetic components (k-mers) associated with motility and biofilm phenotypes in A. xylosoxidans that have biological relevance to these phenotypes in other bacterial species^*a*^

Phenotype	Locus tag	Annotation	Estimated effect	Reference(s)
Motility	AT699_RS2185	Cytochrome *c* oxidase accessory protein CcoG	−14.08 mm	78, 79
	AT699_RS21815	Cytochrome *c* oxidase, *cbb*_3_ type, subunit III	14.98 mm	78, 79
Biofilm	AT699_RS09910	Glycosyltransferase family 1 protein	1.22	80
	AT699_RS20345	Nitrate transport protein NrtA	−2.47	81
	AT699_RS01650	Methionine import ATP-binding protein MetN	1.45	82
	AT699_RS21490	Phosphoglucomutase	1.45	83

a The estimated effect of k-mer presence on the phenotype, the reference for the reported effect, and the annotation of each gene are reported. The full list available in Table S1K to L.

### Increased resistance to meropenem and ceftazidime is established by the direct or indirect regulatory effect of various mutations.

**FIG 5:**
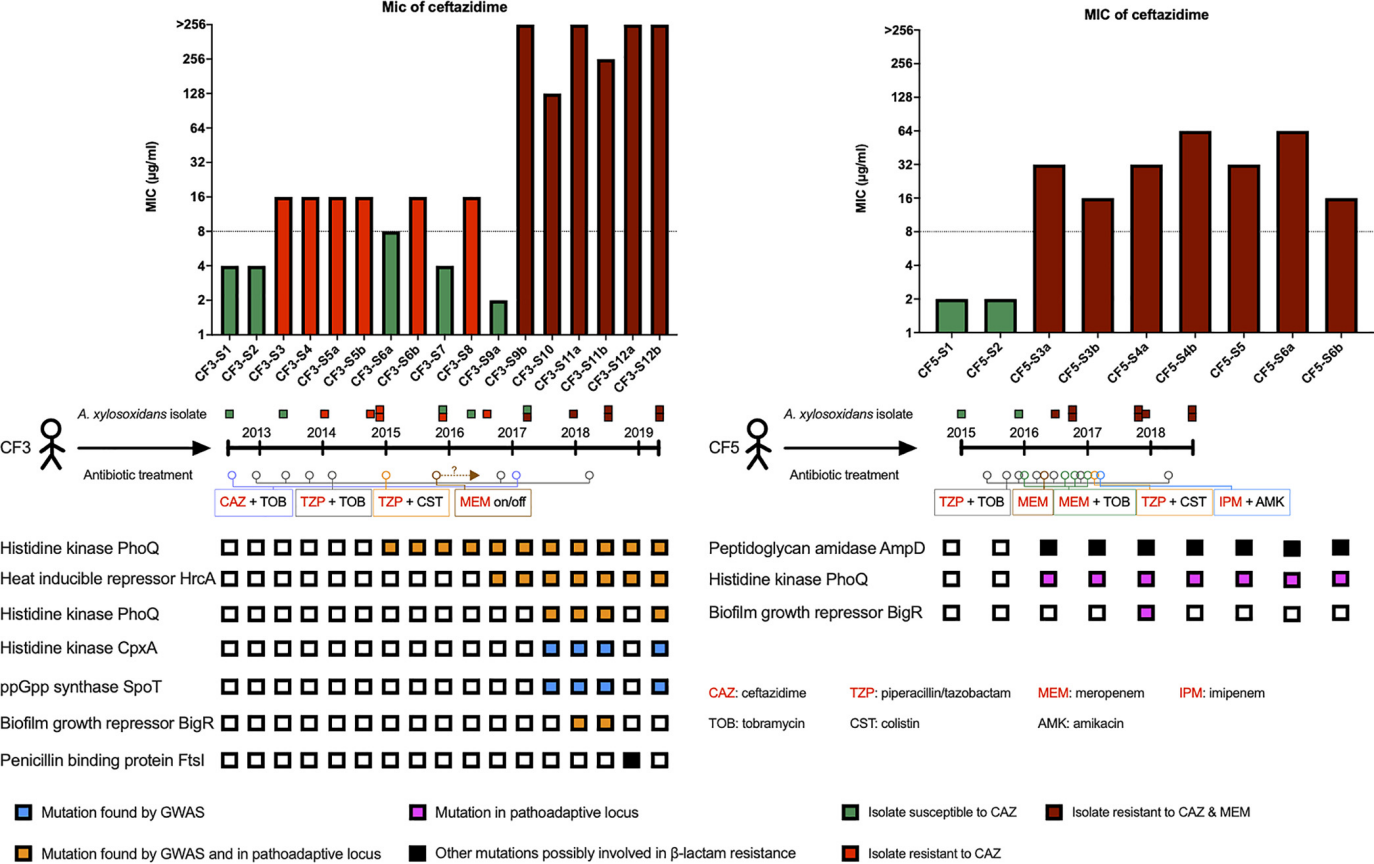
Possible genetic determinants of β-lactam resistance in CF adapted A. xylosoxidans. The top two panels show the measured MICs of the clinical isolates from CF3 and CF5 (Table S1M); the dotted lines represent the considered breakpoints of resistance, and the three bar colors represent different patterns of susceptibility to ceftazidime and/or meropenem. The middle panels represent the timeline of isolates from these two patients and the administered antibiotic combinations based on the clinical data. All β-lactams are in red. The question mark above MEM means that meropenem was used by patient CF3 via inhalation for an unknown period of time. The lower panels represent all genetic changes that may contribute to the increased resistance of ceftazidime and meropenem. Apart from the pathoadaptive gene *hrcA*, all other changes are in putative homologs of previously characterized components of β-lactam resistance in other bacterial species. The presence of the genetic change in each isolate from the topmost panel is denoted by colored squares.

All sequenced isolates of A. xylosoxidans from our collection were resistant to several classes of antibiotics. Based on the clinical data of antimicrobial susceptibility testing (Table S1M), 100% (*n* = 48) of the tested isolates were resistant to aztreonam, 98% (*n* = 54) were resistant to ciprofloxacin, 88% (*n* = 44) were resistant to gentamicin, 87% (*n* = 48) were resistant to tobramycin, 69% (*n* = 37) were resistant to colistin, 47% (*n* = 26) were resistant to ceftazidime, and 33% (*n* = 18) were resistant to meropenem. Furthermore, we found 49 isolates to be multidrug resistant (MDR), i.e., nonsusceptible to at least one agent of three or more classes of antibiotics. As most of the initial isolates were susceptible to ceftazidime and meropenem, we chose to analyze the subsequent isolates from each patient for resistance to these two antibiotics. Notably, there was a consistent increase in resistance to ceftazidime and meropenem in isolates from CF3 and CF5, which was probably selected in response to the regular treatment of these two patients with different β-lactam antibiotics like ceftazidime, meropenem, imipenem, and piperacillin-tazobactam (Fig. 5).

By using GWAS, we found a total of 28 genetic components to be linked to ceftazidime resistance and 5 components associated with meropenem resistance (Table S1N). Most of the components identified represented changes in the genome of isolates from CF3, which may be a result of the higher number of resistant and susceptible isolates from this patient. Moreover, a large number of a total of 33 identified components were in genes encoding signaling proteins (COG T; *n* = 5) or transcription proteins (COG K; *n* = 7). Surprisingly, we also found three of the identified pathoadaptive genes (*phoQ*, *hrcA*, and *bigR*) to be present among the components associated with ceftazidime resistance, suggesting that these regulatory loci are vital for adaptive phenotypes such as antibiotic resistance (Fig. 5). The pathoadaptive gene encoding the putative histidine kinase PhoQ was associated with ceftazidime resistance by two independent genetic components. Mutations in homologs of this gene have an established role for resistance to polymyxin and aminoglycosides in P. aeruginosa (36), but they have also been selected in ceftazidime-treated evolved cultures of S. maltophilia (37). The pathoadaptive gene encoding the biofilm growth-associated repressor BigR has also been shown to have an indirect regulatory effect on antibiotic resistance. The putative homolog of this protein known as ArsR in Enterococcus faecium has been involved in positive regulation of a penicillin-binding protein (PBP), and its deletion has increased resistance to β-lactam antibiotics (38). No information could be obtained for the putative role of *hrcA* homologs in antibiotic resistance, but two other genetic components were found in regulatory encoding genes related to β-lactam resistance (Fig. 5). The first was a gene encoding a putative histidine kinase, CpxA, which has a well-established profile with respect to multidrug antibiotic resistance in several pathogenic bacteria. In this context, the CpxAR two-component response regulator is involved in regulation of the RND (resistance-nodulation-division)-type efflux pumps controlling resistance to β-lactam, aminoglycosides and certain antimicrobial peptides in Escherichia coli, P. aeruginosa, *S*. Typhimurium, Klebsiella pneumoniae, and Vibrio cholerae (39–44). The other component was in the gene encoding the stringent response (p)ppGpp synthase/hydrolase SpoT. Among other functions, (p)ppGpp modulates the expression of PBP or efflux pumps and is linked to β-lactam resistance in other bacterial pathogens (45).

While GWAS may be applicable for unbiased discovery of components linked to antibiotic resistance, it has a statistical limitation for detection of less frequent genetic changes. We therefore looked for other biologically relevant mutations to explain the evolution of β-lactam resistance and found mutations in two genes encoding putative direct effectors of β-lactam resistance in A. xylosoxidans (Fig. 5). In this context, the hypermutator isolate CF3-S12a, which lacked most of the other discussed mutations identified by GWAS, harbored two NS mutations in the gene encoding the penicillin-binding protein FtsI, which is associated with ceftazidime resistance in E. coli (46). Furthermore, in CF5, where a consistent development of resistance in both ceftazidime and meropenem occurred from the third isolate onward, a nonsense mutation was fixed in *ampD*, encoding *N*-acetyl-anhydromuramyl-l-alanine amidase. Inactivation of AmpD has been linked to constitutive overproduction of AmpC β-lactamase and to the rise of β-lactam resistance in most Gram-negative bacterial pathogens (47). Interestingly, we also found another fixed mutation in the gene encoding the pathoadaptive putative histidine kinase PhoQ in the third isolate and all later isolates.

## DISCUSSION

Infections of A. xylosoxidans have recently become more prevalent in patients with CF (5, 6, 9), but there are few studies that characterize the genetic adaptation of *Achromobacter* spp. in CF infections (24, 48, 49). To complement these studies, we used a combination of functional and genomic approaches to reflect on the biology of A. xylosoxidans adaptation in the CF model. In this context, our data set included a larger number of genome-sequenced isolates from each patient to provide a higher resolution of the A. xylosoxidans within-host diversity and capture possible coexisting clades in the different compartments of the CF airway (50). Moreover, we focused exclusively on A. xylosoxidans, because the genetic and phenotypic diversity of different *Achromobacter* species may cause unwanted neglect of A. xylosoxidans specific traits (51, 52). Finally, in addition to the genetic changes, we also investigated the phenotypic changes of A. xylosoxidans isolates and tried to link the genotype with the observed phenotypes. Having multiple isolates per each patient provided an advantage in the analysis of GWAS, where several biologically relevant genetic components were identified for biofilm formation, swimming motility, and β-lactam resistance.

The cystic fibrosis airway represents a complex biological system with multiple environmental stressors affecting adaptation of the colonizing microorganism (53). The differences in composition of infecting pathogens, antibiotic treatments, and immunological host factors impose various selection pressures on adaptation of A. xylosoxidans. This may account for some of the heterogeneity in the genomic characteristics of the six clones isolated from the patients in this study. The hypermutator phenotype was detected in 20% of the isolates (*n* = 11). Hypermutators have been found previously in A. xylosoxidans and other species isolated from CF infection, and it is assumed that the higher mutation rate has a positive effect on faster selection of adaptive phenotypes in this environment (14, 25, 49, 54, 55). It is also likely that this phenotype is fixed in CF1 as a result of its longer colonization in the patient, since it has been suggested that longer evolution history is associated with the hypermutator phenotype (56). Additionally, we observed different signatures of selection between the six clones of A. xylosoxidans that even shifted in one clone (CF3) at different phases of early and late colonization. This demonstrates that A. xylosoxidans has a dynamic adaptation and thrives by natural selection in different infection backgrounds. In this context, negative selection has previously been observed in CF isolates of P. aeruginosa following an initial period of rapid positive selection (57). However, despite these heterogeneities, we still found clear patterns of genetic adaptation of A. xylosoxidans during airway infections in CF.

Our analysis revealed that evolution of regulatory loci plays a major role in within-host evolution of A. xylosoxidans during CF infection. Regulatory proteins often entail pleiotropic functions and are therefore expected to have substantial effects on the overall phenotype development of a bacterial pathogen during infection. We showed that genes encoding signal transduction proteins are the most frequent targets of NS mutations in most of the CF adapted A. xylosoxidans clones. Furthermore, genes encoding other regulatory proteins belonging to the transcription COG category (K) were also under positive selection in at least half of the clones. A possible way to interpret negative or neutral selection of the transcription genes in CF1 and CF3 may be that most of the adaptive NS mutations within these genes are already fixed in the first isolates of these clones as a result of earlier evolution in the CF airway. Previous studies have also shown that mutations in regulatory genes are fixed in the earlier adaptation of P. aeruginosa during CF infections (57). With regard to our patients, we know that CF1 was colonized with A. xylosoxidans for more than 6 years prior to the first isolate included in the study, but to our knowledge there were no previous isolates for CF3. It is possible that this patient had an undetected colonization for some time before the first positive culture, or that the patient was colonized by an evolved CF clone through patient-to-patient transmission (14). However, such a transmission is unlikely to come from patients within the same CF center, as all patients with chronic A. xylosoxidans infection in this study carried their own individual clones. One of the probable transcription genes that could have had preexisting mutations in CF1 is *axyZ*, which was also mutated in isolates of CF2 and CF5. This gene was shown to be under positive selection in other CF-adapted isolates of *Achromobacter* spp. and encodes a transcriptional regulator which represses the AxyXY-OprZ multidrug efflux system in *Achromobacter* spp. (24, 58).

In addition to the enrichment in targets of NS mutations, we showed that all of the pathoadaptive genes in A. xylosoxidans encode regulatory proteins. This finding is also comparable to the enrichment of transcriptional regulators among targets of pathoadaptive genes in CF-adapted P. aeruginosa (18). Moreover, for the first time in studies of *Achromobacter* sp. CF adaptation, we identified two regulatory intergenic loci under positive selection across multiple independent clones. Considering the location of the mutations from these regions, they may regulate expression of the adaptive downstream genes. Previous functional studies emphasized the relevance of these mutations in regulation of adaptive bacterial phenotypes (59, 60).

Finally, with regard to the β-lactam resistance phenotype, several of the components identified by the GWAS analysis were also in genes encoding regulatory proteins. As mentioned in Results, regulatory proteins with established profiles in resistance of other bacterial species, such as SpoT, BigR, CpxA, and PhoQ, are not only regulators of β-lactam resistance. On the contrary, most of these proteins operate within the broader pleiotropic resistance systems by, for example, regulating the transcription of multidrug efflux pumps. In a recent experimental evolution study, it was shown that mutations in genes involved in efflux pump expression were selected in P. aeruginosa in response to combination antibiotic therapy (60). As many of the patients included in our study also received a combination of several different classes of antibiotics, selection of regulatory proteins with pleiotropic resistance effects might be beneficial over selection of direct resistance factor genes. In support of this hypothesis, the last six isolates of CF3, which were resistant to both meropenem and ceftazidime, were also consistently resistant to colistin. Therefore, it is possible that the regulatory mutations in these isolates, among other possible mutations affecting colistin resistance, also had a positive effect on colistin resistance and were selected as a result of antibiotic combination therapy. Beyond the detection of mutations in regulatory protein-encoding genes, finding *ampD* or *ftsI* mutations in association with β-lactam resistance in A. xylosoxidans may seem more trivial. Nevertheless, it highlights the idea that, similar to that in other successful pathogens (61), the evolution of antibiotic resistance in A. xylosoxidans is multifactorial and the product of independent direct or indirect regulatory pathways.

One unique advantage of the k-mer-based GWAS method (DBGWAS) (35) is that it uses the assembled genomes of each isolate and includes variants of all possible accessory genes or mobile genetic elements specifically present in some isolates or clones. Changes within these features are missed by the reference genome-based variant calling. This enabled us to obtain several identified components that were absent in the reference genome. Therefore, this method is not limited to single nucleotide polymorphisms and short insertions or deletions, and more complex genetic changes can be captured. Moreover, we demonstrated that this method associates bacterial genomic with phenotypic evolution for a large collection of mutations and for phenotypes other than antibiotic resistance. However, while the GWAS analysis identified possible causal mutations for the antibiotic resistance, motility, and biofilm phenotypes, it is important to consider it as a preliminary and not a validated result. GWAS was underpowered by the limited number of genomes in our study, and some possible variants with an effect on function may be filtered out because of occurrence in fewer isolates; i.e., we expect that some phenotype-related mutations were not captured by the analysis. We therefore tried to overcome these limitations by focusing only on components of genes with putative established functions in other bacteria. We also performed a manual inspection to find additional mutations not identified by GWAS but with potentially similar putative function based on other studies. To ascertain the real function of these candidate mutations, the mutant alleles have to be constructed in environmental or CF strains of A. xylosoxidans, and the isogenic isolates should be tested for the relevant phenotypes. However, one constraint with the current studies of the pathogen-host adaptation is that the phenotypes are difficult to examine *in vitro* owing to the differences between the infection niche and the *in vitro* conditions. Another caveat for a functional approach is that adaptive regulatory mutations may function in epistatic ways to cause significant effects on adaptive phenotypes (62, 63). Therefore, studying single mutations in one genetic background may not always lead to the optimal result. Future studies considering these challenges can further elucidate the function of the adaptive regulatory mutations identified in this work. Nevertheless, such approaches should also consider the difficulties of generating bacterial genetics in A. xylosoxidans clinical isolates due to lower growth rates and unavailability of antibiotic resistance markers.

**FIG 6:**
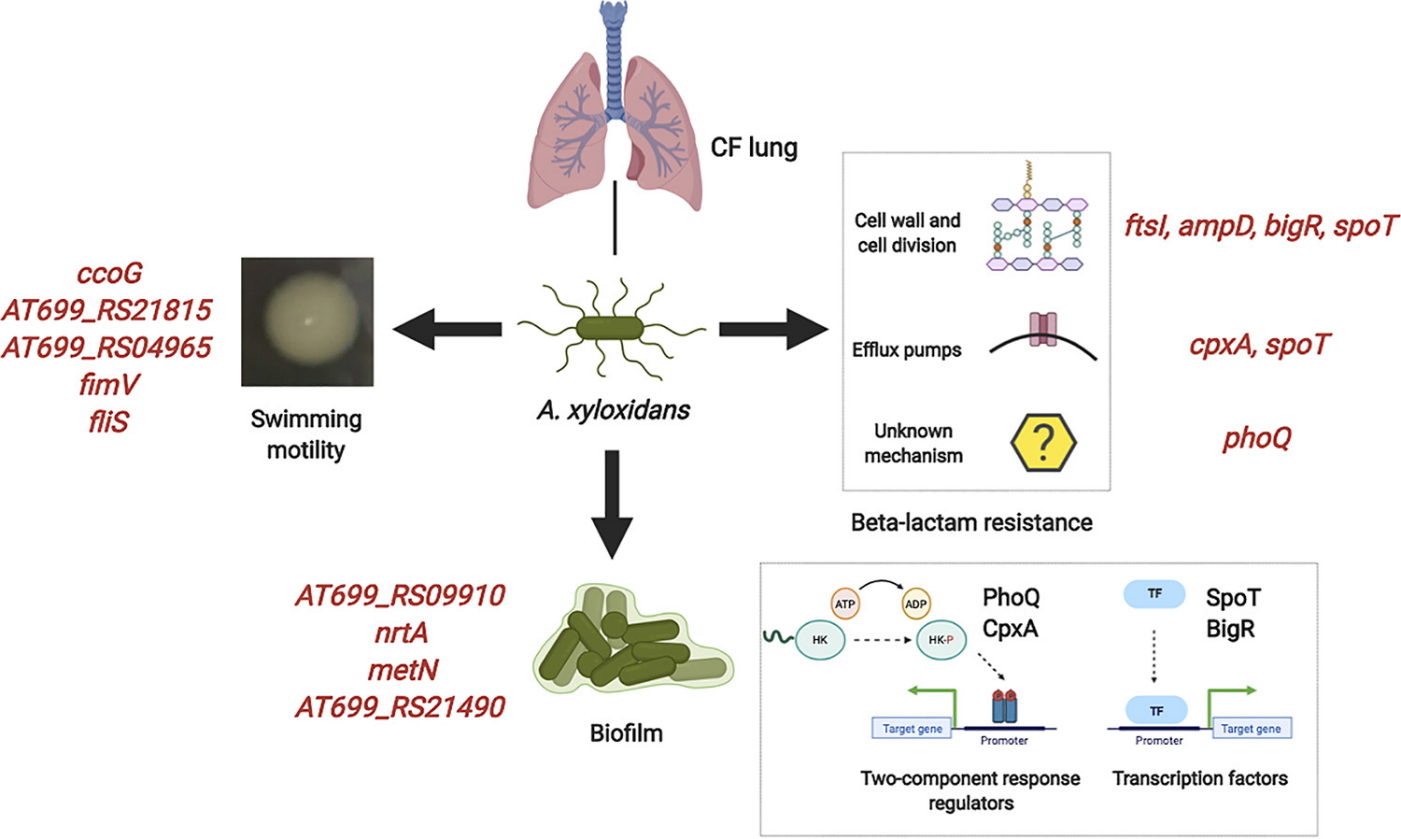
Putative mutations responsible for the adaptive phenotypes of A. xylosoxidans infecting CF airways. The mutations in red were identified by GWAS or manual inspection, where a putative homolog of the loci in other bacterial species have shown similar effects. The lower box shows the function of the four regulatory proteins affected by mutations associated with ceftazidime and meropenem resistance. (Created with BioRender.)

In conclusion, our investigation of the within-host evolution of A. xylosoxidans identified putative genetic determinants for the adaptation of this pathogen in the CF lung (Fig. 6). The analysis identified several regulatory loci under positive selection, and we discuss the likely effect of mutations for the development of the CF adaptive phenotypes. With special focus on the evolution of multidrug antibiotic resistance in this pathogen, we showed that A. xylosoxidans develops further resistance to the few antibiotics to which it was susceptible before colonization. Notably, we found multiple plausible paths for emergence of β-lactam resistance in A. xylosoxidans through mutations in direct or indirect regulatory factors, which may facilitate resistance to other classes of antibiotics used in for CF infection treatment. The knowledge of phenotypic and genetic adaptation of A. xylosoxidans can be vital for advancement of potential therapeutic targets and diagnosis of its infections in patients with CF.

## MATERIALS AND METHODS

### Patient cohort, bacterial strains, and antibiotic susceptibility testing.

Patients with CF registered at the CF center at Skåne University Hospital in Lund and with colonization of A. xylosoxidans in the lower airways were eligible for inclusion in the study. The colonization was defined as chronic in patients where A. xylosoxidans was found in at least half of the sputum cultures for a minimum of 1 year. Clinical isolates from the study participants were obtained from Clinical Microbiology, Labmedicin Skåne, Lund, Sweden. Species identification was done according to standard laboratory methods, including matrix-assisted laser desorption ionization–time of flight mass spectrometry (MALDI-TOF MS) (Bruker Daltonics, Bremen, Germany) (64) and confirmed by sequencing of the *nrdA* locus as previously described (65). Antibiotic susceptibility testing was performed as part of the clinical routine at the department of clinical microbiology according to EUCAST guidelines. Since no breakpoints for resistance of *Achromobacter* spp. have been defined by EUCAST, we considered breakpoints for nonfermenting Gram-negative bacteria as previously described (66). Clinical patient data were obtained from medical records. The study was approved by the Swedish Ethical Review Authority (reference number 2019–02825).

### Genome sequencing, assembly, annotation, and clone type definition.

Genomic DNA was prepared from overnight cultures of bacterial isolates grown on LB medium using a DNeasy blood and tissue kit (Qiagen) and sequenced on an Illumina NextSeq 500 platform generating 150-bp paired-end reads after preparation by the Nextera XT DNA multiplexed protocol at the CTG, Faculty of Medicine, Lund University, Sweden. This resulted in an average of 1,610,153 reads (range, 1,019,699 to 2,854,331) for each of the genomic libraries. The assembly of the sequence reads from each isolate was performed by SPAdes version 3.10.1 (67) with default parameters and k-mer sizes ranging from 21 to 127. The average assembly size was 213 contigs (range, 171 to 270). The assembled contigs were annotated using Prokka (68) using the NCTC10807 reference genome (NCBI accession number PRJEB6403) as the first annotation priority. To analyze the genetic distance between isolates of the six patients, multilocus sequence typing (MLST) analysis was performed on the genome of each lineage using the MLST database and as previously described (65).

### Genome alignment, variant calling, and phylogenetic trees.

Genome alignment and within-host introduced variants were called with BacDist (https://github.com/MigleSur/BacDist) (24, 69), which is based on snippy variant calling (70), and filtering of variants present in all samples, using NCTC10807 as a reference genome with default parameters. Hypermutator phenotype was checked by inspection of the BacDist variants and manual search of additional deletion or insertions in the A. xylosoxidans DNA mismatch repair system genes as previously described (49). The within-host-introduced variants (Table S2) were used for phylogenetic tree generation with RAxML (71) using the GTRCAT model.

### Substitution rate estimation and signatures of selection.

The substitution rate for bacterial isolates was estimated for all lineages using BEAUti (BEAST version 2.5.0) (72). The Markov chain Monte Carlo (MCMC) was run for 50,000,000 iterations with the sequence alignments from BacDist as the input and using the HKY substitution model, strict clock parameters, coalescent constant population tree prior, 1/X prior for population size, and gamma prior for the clock rate. Tracer software (version 1.7.1) (73) was used to control convergence in an effective sample size and parameter value traces. Multiple tests were performed for each sample to confirm reproducibility and convergence. The calculated clock rate (per site per year) was multiplied by the total size of the alignment to get the substitution rate per genome per year. Signature of selection (*dN*/*dS*) was calculated by division of the total number of nonsynonymous mutations by the total number of synonymous mutations, which had been multiplied by three, since the number of nonsynonymous sites is approximately three times higher than the number of synonymous sites in other similar bacterial genomes (57). Statistical significance for higher or lower *dN*/*dS* for any clone was calculated by two-tailed Fisher’s exact using the number of S and NS mutations of that clone compared to that of all remaining clones.

### Analysis of the COG groups.

The reference genome NCTC10807 and the list of variants (Table S2) were annotated using EGGNOG-mapper version 1.0.3 (74) at the DIAMOND, eggNOG’s bacterial database, with a 1 × 10^−8^ E value cutoff and 0.8 minimum query sequence coverage settings. Within each of the 21 identified COG targets, the number of NS mutations was counted and divided by the sum to get the percentage for each COG among NS mutations. This value was divided by the percentage for each COG in the NCTC10807 reference genome to obtain the enrichments of each group. Statistical significance for enrichment of each group in the data set was calculated by the two-tailed Fisher’s exact test and adjustment of the raw *P* value using the Bonferroni method.

### Identification of genetic loci under positive selection.

The A. xylosoxidans reference genome NCTC10807 with the annotated coding sequence regions was retrieved from the NCBI database. The total observed number of mutations in each coding or noncoding locus was recorded for each clone (Table S4). To identify loci under positive selection across different clones (convergent evolution), each locus with more than one mutation in the same clone was considered to have one clonal mutation. The number of clonal NS mutations in each clone (Table S1D) was distributed across the 6,214 genes based on the length of each gene divided by the total size of the coding region, where longer genes are expected to acquire more mutations than shorter ones (Table S5). The sum of the expected value of mutations in each gene across the 6 clones was compared with that of the observed number of mutations. Genes mutated in at least half of the clones (3 clones), with an observed versus expected enrichment of more than 10-fold, and a statistically significant difference in mutation density [Poisson, *P*(*x*;μ) < 0.0001, where *x* is the observed mutation number and μ is the expected mutation number], were defined as genes under positive selection for adaptation, i.e., pathoadaptive genes. Similarly, for identification of pathoadaptive noncoding loci mutations, the same method was applied but with the additional constraint that the clonal mutations had to be within close proximity, i.e., no more than 35 bp apart, similar to a previously described method (59).

10.1128/mSystems.00523-21.4 TABLE S4 Count of nonsynonymous or intergenic mutations in the coding or noncoding regions of the reference genome among isolates of each clone. Download Table S4, XLSX file, 1.3 MB. Copyright © 2021 Khademi et al. 2021 Khademi et al. https://creativecommons.org/licenses/by/4.0/ This content is distributed under the terms of the Creative Commons Attribution 4.0 International license.

10.1128/mSystems.00523-21.5 TABLE S5 Expected and observed counts of clonal mutations in each coding and noncoding region of the reference genome based on Table S4. Pathoadaptive loci are highlighted in red. Download Table S5, XLSX file, 2.9 MB. Copyright © 2021 Khademi et al. 2021 Khademi et al. https://creativecommons.org/licenses/by/4.0/ This content is distributed under the terms of the Creative Commons Attribution 4.0 International license.

### Swimming motility, growth rate, and biofilm formation assays.

ABTGC minimal medium (75) consisting of 2 g/liter (NH4)_2_SO_4_, 6 g/liter Na_2_HPO_4_, 3 g/liter KH_2_PO_4_, 3 g/liter NaCl, 1 mM MgCl_2_, 0.1 mM CaCl_2_, 0.01 mM FeCl_3_, 2.5 mg/liter thiamine supplemented with 0.5% (wt/vol) glucose, and Casamino Acids (Merck), was used during all phenotype assays and to make overnight cultures. To measure swimming motility of A. xylosoxidans, a similar-sized bacterial colony of each isolate was spotted in the middle of an ABTGC 0.3% (wt/vol) agar plate. The plates were incubated at 37°C for 24 h, and the diameters of migration from the spot were recorded. The growth experiments were performed in 96-well microtiter plates (Thermo Scientific). Briefly, the overnight cultures from each isolate were diluted to an OD_600_ of 0.1, and 15 μl was added in a total volume of 150 μl in three wells of the plates. Bacterial growth (OD_600_) was measured continuously for 22 h at 37°C with shaking using a SpectraMax multimode reader (Molecular Devices). The growth data were analyzed with Microsoft Excel (Microsoft), where the doubling time of each strain was calculated during exponential growth.

Biofilm formation was also analyzed in 96-well microtiter plates using crystal violet staining. Each overnight culture was diluted to an OD_600_ of 0.5 and inoculated by addition of 18 μl to a total volume of 180 μl in each well. Medium alone (180 μl) was used as the negative control. The plate was incubated for 48 h at 37°C and 125 rpm shaking. Thereafter, the bacterial broth was discarded, the wells were washed three times with 200 μl of phosphate-buffered saline (PBS), and the biomass in the wells was fixed by addition of 200 μl methanol for 10 min. After removal of the methanol, the wells were allowed to dry for 2 h and then stained with 160 μl of 0.1% (wt/vol) crystal violet for 4 min, followed by 3 washes with 200 μl PBS to remove the excess free crystal violet. The crystal violet was solubilized with acetone-ethanol (4:1 [vol/vol]) and quantified at OD_550_ using SpectraMax multimode reader. Each experiment was performed in triplicate and repeated by three biological replicates. For each measurement, one-way analysis of variance (ANOVA) followed by Tukey’s honestly significant difference (HSD) multiple-comparison test using GraphPad Prism 9 was used to calculate the significance of change in the mean from the earliest isolates of each patient. The adjusted *P* value of <0.05 was used for significance. Isolates with mean OD_550_ values greater than three times that of the negative control were considered biofilm producers (76).

### Identification of genetic loci associated with motility, biofilm, growth, and antibiotic resistance.

The significant genetic components associated with motility, growth, biofilm, ceftazidime, and meropenem resistance were identified using the DBGWAS version 0.5.4 software (34). The cutoff for ceftazidime and meropenem resistance was defined as described above. The other cutoff values were defined as motility of >10 mm, doubling time of >100 min, and biofilm OD_550_ of >0.3. The *de novo* assembled contigs of the 55 isolates were used as input in the software. All available annotations of *Achromobacter* genes from the UniProt database (77) were used for unitig annotations (271,851 genes; retrieved 17 April 2020) for motility, doubling time, and biofilm GWAS analysis; all known bacterial resistance genes from the UniProt database (76) were used for unitig annotations (7,792 genes; retrieved 17 April 2020) in ceftazidime and meropenem resistance GWAS. All identified components with *q* value of <0.05 were investigated further. In every component with more than one significant node, the node with the lowest *q* value was considered. Every k-mer present in only one isolate was discarded to avoid any possible phenotype bias.

### Data availability.

DNA sequence reads from the 55 A. xylosoxidans isolates are available in the European Nucleotide Archive under study accession number PRJEB43175.

## Supplementary Material

Reviewer comments

## References

[1] Didelot X, Walker AS, Peto TE, Crook DW, Wilson DJ. 2016. Within-host evolution of bacterial pathogens. Nat Rev Microbiol 14:150–162. doi:10.1038/nrmicro.2015.13. 26806595 PMC5053366

[2] Ciofu O, Hansen CR, Høiby N. 2013. Respiratory bacterial infections in cystic fibrosis. Curr Opin Pulm Med 19:251–258. doi:10.1097/MCP.0b013e32835f1afc. 23449384

[3] Tetart M, Wallet F, Kyheng M, Leroy S, Perez T, Le Rouzic O, Wallaert B, Prevotat A. 2019. Impact of Achromobacter xylosoxidans isolation on the respiratory function of adult patients with cystic fibrosis. ERJ Open Res 5:e00051-19. doi:10.1183/23120541.00051-2019. PMC6899338 31832429

[4] Qvist T, Taylor-Robinson D, Waldmann E, Olesen HV, Hansen CR, Mathiesen IH, Høiby N, Katzenstein TL, Smyth RL, Diggle PJ, Pressler T. 2016. Comparing the harmful effects of nontuberculous mycobacteria and Gram negative bacteria on lung function in patients with cystic fibrosis. J Cyst Fibros 15:380–385. doi:10.1016/j.jcf.2015.09.007. 26482717 PMC4893021

[5] Ridderberg W, Bendstrup KEM, Olesen HV, Jensen-Fangel S, Nørskov-Lauritsen N. 2011. Marked increase in incidence of Achromobacter xylosoxidans infections caused by sporadic acquisition from the environment. J Cyst Fibros 10:466–469. doi:10.1016/j.jcf.2011.07.004. 21835703

[6] Trancassini M, Iebba V, Citera N, Tuccio V, Magni A, Varesi P, De Biase RV, Totino V, Santangelo F, Gagliardi A, Schippa S. 2014. Outbreak of achromobacter xylosoxidans in an italian cystic fibrosis center: genome variability, biofilm production, antibiotic resistance, and motility in isolated strains. Front Microbiol 5:1–8. doi:10.3389/fmicb.2014.00138. 24772108 PMC3982067

[7] De Baets F, Schelstraete P, Van Daele S, Haerynck F, Vaneechoutte M. 2007. Achromobacter xylosoxidans in cystic fibrosis: prevalence and clinical relevance. J Cyst Fibros 6:75–78. doi:10.1016/j.jcf.2006.05.011. 16793350

[8] Spicuzza L, Sciuto C, Vitaliti G, Dio G, Leonardi S, Rosa M. 2009. Emerging pathogens in cystic fibrosis: ten years of follow-up in a cohort of patients. Eur J Clin Microbiol Infect Dis 28:191–195. doi:10.1007/s10096-008-0605-4. 18758832

[9] Razvi S, Quittell L, Sewall A, Quinton H, Marshall B, Saiman L. 2009. Respiratory microbiology of patients with cystic fibrosis in the United States, 1995 to 2005. Chest 136:1554–1560. doi:10.1378/chest.09-0132. 19505987

[10] Duggan JM, Goldstein SJ, Chenoweth CE, Kauffman CA, Bradley SF. 1996. Achromobacter xylosoxidans bacteremia: report of four cases and review of the literature. Clin Infect Dis 23:569–576. doi:10.1093/clinids/23.3.569. 8879782

[11] Yabuuchi E, Oyama A. 1971. Achromobacter xylosoxidans n. sp. from human ear discharge. Jpn J Microbiol 15:477–481. doi:10.1111/j.1348-0421.1971.tb00607.x. 5316576

[12] Aisenberg G, Rolston KV, Safdar A. 2004. Bacteremia caused by Achromobacter and Alcaligenes species in 46 patients with cancer (1989–2003.). Cancer 101:2134–2140. doi:10.1002/cncr.20604. 15389476

[13] Gómez-Cerezo J. 2003. Achromobacter xylosoxidans bacteremia: a 10-year analysis of 54 cases. Eur J Clin Microbiol Infect Dis 22:360–363. doi:10.1007/s10096-003-0925-3. 12750959

[14] Gabrielaite M, Bartell JA, Nørskov-Lauritsen N, Pressler T, Nielsen FC, Johansen HK, Marvig RL. 2021. Transmission and antibiotic resistance of Achromobacter in cystic fibrosis. J Clin Microbiol 59:1–12. doi:10.1128/JCM.02911-20. PMC8092725 33472899

[15] Glupczynski Y, Hansen W, Freney J, Yourassowsky E. 1988. In vitro susceptibility of Alcaligenes denitrificans subsp. xylosoxidans to 24 antimicrobial agents. Antimicrob Agents Chemother 32:276–278. doi:10.1128/AAC.32.2.276. 3163242 PMC172153

[16] Van Daele S, Verhelst R, Claeys G, Verschraegen G, Franckx H, Van Simaey L, de Ganck C, De Baets F, Vaneechoutte M. 2005. Shared genotypes of Achromobacter xylosoxidans strains isolated from patients at a cystic fibrosis rehabilitation center. J Clin Microbiol 43:2998–3002. doi:10.1128/JCM.43.6.2998-3002.2005. 15956444 PMC1151887

[17] Lambiase A, Catania MR, del Pezzo M, Rossano F, Terlizzi V, Sepe A, Raia V. 2011. Achromobacter xylosoxidans respiratory tract infection in cystic fibrosis patients. Eur J Clin Microbiol Infect Dis 30:973–980. doi:10.1007/s10096-011-1182-5. 21279730 PMC3132409

[18] Marvig RL, Sommer LM, Molin S, Johansen HK. 2015. Convergent evolution and adaptation of Pseudomonas aeruginosa within patients with cystic fibrosis. Nat Genet 47:57–64. doi:10.1038/ng.3148. 25401299

[19] Bernardy EE, Petit RA, Raghuram V, Alexander AM, Read TD, Goldberg JB. 2020. Genotypic and phenotypic diversity of staphylococcus aureus isolates from cystic fibrosis patient lung infections and their interactions with Pseudomonas aeruginosa. mBio 11:e00735-20. doi:10.1128/mBio.00735-20. 32576671 PMC7315118

[20] Esposito A, Pompilio A, Bettua C, Crocetta V, Giacobazzi E, Fiscarelli E, Jousson O, Di Bonaventura G. 2017. Evolution of stenotrophomonas maltophilia in cystic fibrosis lung over chronic infection: a genomic and phenotypic population study. Front Microbiol 8:1590. doi:10.3389/fmicb.2017.01590. 28894437 PMC5581383

[21] Lieberman TD, Michel J-B, Aingaran M, Potter-Bynoe G, Roux D, Davis MR, Skurnik D, Leiby N, LiPuma JJ, Goldberg JB, McAdam AJ, Priebe GP, Kishony R. 2011. Parallel bacterial evolution within multiple patients identifies candidate pathogenicity genes. Nat Genet 43:1275–1280. doi:10.1038/ng.997. 22081229 PMC3245322

[22] Silva IN, Santos PM, Santos MR, Zlosnik JEA, Speert DP, Buskirk SW, Bruger EL, Waters CM, Cooper VS, Moreira LM. 2016. Long-term evolution of Burkholderia multivorans during a chronic cystic fibrosis infection reveals shifting forces of selection. mSystems 1:e00029-16. doi:10.1128/mSystems.00029-16. 27822534 PMC5069766

[23] Miller JH. 1996. Spontaneous mutators in bacteria: insights into pathways of mutagenesis and repair. Annu Rev Microbiol 50:625–643. doi:10.1146/annurev.micro.50.1.625. 8905093

[24] Gabrielaite M, Nørskov-Lauritsen N, Nielsen FC, Marvig RL. 2020. Achromobacter genetic adaptation in cystic fibrosis. bioRxiv 2020.08.04.235952. 10.1099/mgen.0.000582 PMC8477396 34232117

[25] Marvig RL, Johansen HK, Molin S, Jelsbak L. 2013. Genome analysis of a transmissible lineage of pseudomonas aeruginosa reveals pathoadaptive mutations and distinct evolutionary paths of hypermutators. PLoS Genet 9:e1003741. doi:10.1371/journal.pgen.1003741. 24039595 PMC3764201

[26] Kryazhimskiy S, Plotkin JB. 2008. The population genetics of *dN*/*dS*. PLoS Genet 4:e1000304. doi:10.1371/journal.pgen.1000304. 19081788 PMC2596312

[27] Guimarães BG, Barbosa RL, Soprano AS, Campos BM, de Souza TA, Tonoli CCC, Leme AFP, Murakami MT, Benedetti CE. 2011. Plant pathogenic bacteria utilize biofilm growth-associated repressor (BigR), a novel winged-helix redox switch, to control hydrogen sulfide detoxification under hypoxia. J Biol Chem 286:26148–26157. doi:10.1074/jbc.M111.234039. 21632538 PMC3138302

[28] Walsh BJC, Wang J, Edmonds KA, Palmer LD, Zhang Y, Trinidad JC, Skaar EP, Giedroc DP. 2020. The response of acinetobacter baumannii to hydrogen sulfide reveals two independent persulfide-sensing systems and a connection to biofilm regulation. mBio 11:e01254-20. doi:10.1128/mBio.01254-20. 32576676 PMC7315123

[29] Miller SI, Kukral AM, Mekalanos JJ. 1989. A two-component regulatory system (phoP phoQ) controls Salmonella typhimurium virulence. Proc Natl Acad Sci U S A 86:5054–5058. doi:10.1073/pnas.86.13.5054. 2544889 PMC297555

[30] Adams P, Fowler R, Kinsella N, Howell G, Farris M, Coote P, O'Connor CD. 2001. Proteomic detection of PhoPQ- and acid-mediated repression of Salmonella motility. Proteomics 1:597–607. doi:10.1002/1615-9861(200104)1:4<597::AID-PROT597>3.0.CO;2-P. 11681212

[31] McPhee JB, Lewenza S, Hancock REW. 2003. Cationic antimicrobial peptides activate a two-component regulatory system, PmrA-PmrB, that regulates resistance to polymyxin B and cationic antimicrobial peptides in Pseudomonas aeruginosa. Mol Microbiol 50:205–217. doi:10.1046/j.1365-2958.2003.03673.x. 14507375

[32] Visca P, Imperi F, Lamont IL. 2007. Pyoverdine siderophores: from biogenesis to biosignificance. Trends Microbiol 15:22–30. doi:10.1016/j.tim.2006.11.004. 17118662

[33] Hermansen GMM, Hansen ML, Khademi SMH, Jelsbak L. 2018. Intergenic evolution during host adaptation increases expression of the metallophore pseudopaline in Pseudomonas aeruginosa. Microbiology (Reading) 164:1038–1047. doi:10.1099/mic.0.000687. 29969091

[34] Schick A, Kassen R. 2018. Rapid diversification of Pseudomonas aeruginosa in cystic fibrosis lung-like conditions. Proc Natl Acad Sci U S A 115:10714–10719. doi:10.1073/pnas.1721270115. 30275334 PMC6196507

[35] Jaillard M, Lima L, Tournoud M, Mahé P, van Belkum A, Lacroix V, Jacob L. 2018. A fast and agnostic method for bacterial genome-wide association studies: bridging the gap between k-mers and genetic events. PLoS Genet 14:e1007758. doi:10.1371/journal.pgen.1007758. 30419019 PMC6258240

[36] Macfarlane ELA, Kwasnicka A, Hancock REW. 2000. Role of Pseudomonas aeruginosa Phop-PhoQ in resistance to antimicrobial cationic peptides and aminoglycosides. Microbiology 146:2543–2554. doi:10.1099/00221287-146-10-2543. 11021929

[37] Blanco P, Corona F, Martínez JL. 2019. Involvement of the RND efflux pump transporter SmeH in the acquisition of resistance to ceftazidime in Stenotrophomonas maltophilia. Sci Rep 9:4917. doi:10.1038/s41598-019-41308-9. 30894628 PMC6426872

[38] Lebreton F. 2012. AsrR is an oxidative stress sensing regulator modulating Enterococcus faecium opportunistic traits, antimicrobial resistance, and pathogenicity. PLoS Pathog 8:e1002834. doi:10.1371/journal.ppat.1002834. 22876178 PMC3410868

[39] Hirakawa H, Nishino K, Yamada J, Hirata T, Yamaguchi A. 2003. β-Lactam resistance modulated by the overexpression of response regulators of two-component signal transduction systems in Escherichia coli. J Antimicrob Chemother 52:576–582. doi:10.1093/jac/dkg406. 12951338

[40] Kohanski MA, Dwyer DJ, Wierzbowski J, Cottarel G, Collins JJ. 2008. Mistranslation of membrane proteins and two-component system activation trigger antibiotic-mediated cell death. Cell 135:679–690. doi:10.1016/j.cell.2008.09.038. 19013277 PMC2684502

[41] Tian ZX, Yi XX, Cho A, O’Gara F, Wang YP. 2016. CpxR activates MexAB-OprM efflux pump expression and enhances antibiotic resistance in both laboratory and clinical nalB-type isolates of Pseudomonas aeruginosa. PLoS Pathog 12:e1005932. doi:10.1371/journal.ppat.1005932. 27736975 PMC5063474

[42] Huang H, Sun Y, Yuan L, Pan Y, Gao Y, Ma C, Hu G. 2016. Regulation of the two-component regulator CpxR on aminoglycosides and β-lactams resistance in Salmonella enterica serovar typhimurium. Front Microbiol 7:1–10. doi:10.3389/fmicb.2016.00604. 27199934 PMC4846824

[43] Srinivasan VB, Vaidyanathan V, Mondal A, Rajamohan G. 2012. Role of the two component signal transduction system CPxAR in conferring cefepime and chloramphenicol resistance in Klebsiella pneumoniae NTUH-K2044. PLoS One 7:e33777. doi:10.1371/journal.pone.0033777. 22496764 PMC3319533

[44] Taylor DL, Renee Bina X, Slamti L, Waldor MK, Bina JE. 2014. Reciprocal regulation of resistance-nodulation-division efflux systems and the Cpx two-component system in vibrio cholerae. Infect Immun 82:2980–2991. doi:10.1128/IAI.00025-14. 24799626 PMC4097637

[45] Das B, Bhadra RK. 2020. (p)ppGpp metabolism and antimicrobial resistance in bacterial pathogens. Front Microbiol 11:563944. doi:10.3389/fmicb.2020.563944. 33162948 PMC7581866

[46] Tavío MM, Aquili VD, Vila J, Poveda JB. 2014. Resistance to ceftazidime in Escherichia coli associated with AcrR, MarR and PBP3 mutations and overexpression of sdiA. J Med Microbiol 63:56–65. doi:10.1099/jmm.0.063727-0. 24089577

[47] Thomson KS, Smith Moland E. 2000. Version 2000: the new β-lactamases of Gram-negative bacteria at the dawn of the new millennium. Microbes Infect 2:1225–1235. doi:10.1016/S1286-4579(00)01276-4. 11008112

[48] Ridderberg W, Nielsen SM, Nørskov-Lauritsen N. 2015. Genetic adaptation of achromobacter sp. during persistence in the lungs of cystic fibrosis patients. PLoS One 10:e0136790. doi:10.1371/journal.pone.0136790. 26313451 PMC4552427

[49] Veschetti L, Sandri A, Johansen HK, Lleò MM, Malerba G. 2020. Hypermutation as an evolutionary mechanism for achromobacter xylosoxidans in cystic fibrosis lung infection. Pathogens 9:72. doi:10.3390/pathogens9020072. PMC7168687 31973169

[50] Jorth P, Staudinger BJ, Wu X, Hisert KB, Hayden H, Garudathri J, Harding CL, Radey MC, Rezayat A, Bautista G, Berrington WR, Goddard AF, Zheng C, Angermeyer A, Brittnacher MJ, Kitzman J, Shendure J, Fligner CL, Mittler J, Aitken ML, Manoil C, Bruce JE, Yahr TL, Singh PK. 2015. Regional isolation drives bacterial diversification within cystic fibrosis lungs. Cell Host Microbe 18:307–319. doi:10.1016/j.chom.2015.07.006. 26299432 PMC4589543

[51] Filipic B, Malesevic M, Vasiljevic Z, Lukic J, Novovic K, Kojic M, Jovcic V. 2017. Uncovering differences in virulence markers associated with Achromobacter species of CF and non-CF origin. Front Cell Infect Microbiol 7:224. doi:10.3389/fcimb.2017.00224. 28611955 PMC5447083

[52] Jeukens J, Freschi L, Vincent AT, Emond-Rheault J-G, Kukavica-Ibrulj I, Charette SJ, Levesque RC. 2017. A pan-genomic approach to understand the basis of host adaptation in achromobacter. Genome Biol Evol 9:1030–1046. doi:10.1093/gbe/evx061. 28383665 PMC5405338

[53] Folkesson A, Jelsbak L, Yang L, Johansen HK, Ciofu O, Høiby N, Molin S. 2012. Adaptation of Pseudomonas aeruginosa to the cystic fibrosis airway: an evolutionary perspective. Nat Rev Microbiol 10:841–851. doi:10.1038/nrmicro2907. 23147702

[54] Watson ME, Burns JL, Smith AL. 2004. Hypermutable Haemophilus influenzae with mutations in mutS are found in cystic fibrosis sputum. Microbiology (Reading) 150:2947–2958. doi:10.1099/mic.0.27230-0. 15347753

[55] Prunier A‐L, Malbruny B, Laurans M, Brouard J, Duhamel J‐F, Leclercq R. 2003. High rate of macrolide resistance in Staphylococcus aureus strains from patients with cystic fibrosis reveals high proportions of hypermutable strains. J Infect Dis 187:1709–1716. doi:10.1086/374937. 12751028

[56] Ciofu O, Riis B, Pressler T, Poulsen HE, Høiby N. 2005. Occurrence of hypermutable Pseudomonas aeruginosa in cystic fibrosis patients is associated with the oxidative stress caused by chronic lung inflammation. Antimicrob Agents Chemother 49:2276–2282. doi:10.1128/AAC.49.6.2276-2282.2005. 15917521 PMC1140492

[57] Yang L, Jelsbak L, Marvig RL, Damkiaer S, Workman CT, Rau MH, Hansen SK, Folkesson A, Johansen HK, Ciofu O, Hoiby N, Sommer MOA, Molin S. 2011. Evolutionary dynamics of bacteria in a human host environment. Proc Natl Acad Sci U S A 108:7481–7486. doi:10.1073/pnas.1018249108. 21518885 PMC3088582

[58] Bador J, Neuwirth C, Grangier N, Muniz M, Germé L, Bonnet J, Pillay V-G, Llanes C, de Curraize C, Amoureux L. 2017. Role of AxyZ transcriptional regulator in overproduction of AxyXY-OprZ multidrug efflux system in Achromobacter species mutants selected by tobramycin. Antimicrob Agents Chemother 61:e00290-17. doi:10.1128/AAC.00290-17. 28584156 PMC5527583

[59] Khademi SMH, Sazinas P, Jelsbak L. 2019. Within-host adaptation mediated by intergenic evolution in Pseudomonas aeruginosa. Genome Biol Evol 11:1385–1397. doi:10.1093/gbe/evz083. 30980662 PMC6505451

[60] Barbosa C, Mahrt N, Bunk J, Graßer M, Rosenstiel P, Jansen G, Schulenburg H. 2020. The genomic basis of rapid adaptation to antibiotic combination therapy in Pseudomonas aeruginosa. Mol Biol Evol 38:449–464. doi:10.1093/molbev/msaa233. PMC7826179 32931584

[61] Jorth P, McLean K, Ratjen A, Secor PR, Bautista GE, Ravishankar S, Rezayat A, Garudathri J, Harrison JJ, Harwood RA, Penewit K, Waalkes A, Singh PK, Salipante SJ. 2017. Evolved aztreonam resistance is multifactorial and can produce hypervirulence in pseudomonas aeruginosa. mBio 8:e00517-17. doi:10.1128/mBio.00517-17. 29089424 PMC5666152

[62] Jochumsen N, Marvig RL, Damkiær S, Jensen RL, Paulander W, Molin S, Jelsbak L, Folkesson A. 2016. The evolution of antimicrobial peptide resistance in Pseudomonas aeruginosa is shaped by strong epistatic interactions. Nat Commun 7:13002. doi:10.1038/ncomms13002. 27694971 PMC5494192

[63] Damkiær S, Yang L, Molin S, Jelsbak L. 2013. Evolutionary remodeling of global regulatory networks during long-term bacterial adaptation to human hosts. Proc Natl Acad Sci U S A 110:7766–7771. doi:10.1073/pnas.1221466110. 23610385 PMC3651418

[64] Jorgensen JH, Pfaller MA, Carroll KC, Funke G, Landry ML, Richter S, Warnock DW (ed). 2015. Manual of clinical microbiology, 11th ed. ASM Press, Washington, DC.

[65] Spilker T, Vandamme P, LiPuma JJ. 2012. A multilocus sequence typing scheme implies population structure and reveals several putative novel achromobacter species. J Clin Microbiol 50:3010–3015. doi:10.1128/JCM.00814-12. 22785192 PMC3421806

[66] Barragán EP, Pérez JS, Corbella L, Orellana MÁ, Fernández-Ruiz M. 2018. Achromobacter xylosoxidans bacteremia: clinical and microbiological features in a 10-year case series. Rev Esp Quimioter 31:268–273. 29806765 PMC6166261

[67] Bankevich A, Nurk S, Antipov D, Gurevich AA, Dvorkin M, Kulikov AS, Lesin VM, Nikolenko SI, Pham S, Prjibelski AD, Pyshkin AV, Sirotkin AV, Vyahhi N, Tesler G, Alekseyev MA, Pevzner PA. 2012. SPAdes: a new genome assembly algorithm and its applications to single-cell sequencing. J Comput Biol 19:455–477. doi:10.1089/cmb.2012.0021. 22506599 PMC3342519

[68] Seemann T. 2014. Prokka: rapid prokaryotic genome annotation. Bioinformatics 30:2068–2069. doi:10.1093/bioinformatics/btu153. 24642063

[69] Gabrielaite M, Misiakou M-A, Marvig RL. 2020. BacDist: Snakemake pipeline for bacterial SNP distance and phylogeny analysis. doi:10.5281/zenodo.3667680.

[70] Seemann T. 2018. Snippy: rapid haploid variant calling and core genome alignment. https://github.com/tseemann/snippy.

[71] Stamatakis A. 2014. RAxML version 8: a tool for phylogenetic analysis and post-analysis of large phylogenies. Bioinformatics 30:1312–1313. doi:10.1093/bioinformatics/btu033. 24451623 PMC3998144

[72] Bouckaert R, Heled J, Kühnert D, Vaughan T, Wu C-H, Xie D, Suchard MA, Rambaut A, Drummond AJ. 2014. BEAST 2: a software platform for Bayesian evolutionary analysis. PLoS Comput Biol 10:e1003537. doi:10.1371/journal.pcbi.1003537. 24722319 PMC3985171

[73] Rambaut A, Drummond AJ, Xie D, Baele G, Suchard MA. 2018. Posterior summarization in Bayesian phylogenetics using Tracer 1.7. Syst Biol 67:901–904. doi:10.1093/sysbio/syy032. 29718447 PMC6101584

[74] Huerta-Cepas J, Forslund K, Coelho LP, Szklarczyk D, Jensen LJ, von Mering C, Bork P. 2017. Fast genome-wide functional annotation through orthology assignment by eggNOG-mapper. Mol Biol Evol 34:2115–2122. doi:10.1093/molbev/msx148. 28460117 PMC5850834

[75] Marvig RL, Damkiær S, Khademi SMH, Markussen TM, Molin S, Jelsbak L. 2014. Within-host evolution of pseudomonas aeruginosa reveals adaptation toward iron acquisition from hemoglobin. mBio 5:e00966-14. doi:10.1128/mBio.00966-14. 24803516 PMC4010824

[76] Stepanović S, Vuković D, Hola V, Di Bonaventura G, Djukić S, Cirković I, Ruzicka F. 2007. Quantification of biofilm in microtiter plates: overview of testing conditions and practical recommendations for assessment of biofilm production by staphylococci. APMIS 115:891–899. doi:10.1111/j.1600-0463.2007.apm_630.x. 17696944

[77] The UniProt Consortium. 2019. UniProt: a worldwide hub of protein knowledge. Nucleic Acids Res 47:D506–D515. doi:10.1093/nar/gky1049. 30395287 PMC6323992

[78] van der Stel A-X, Boogerd FC, Huynh S, Parker CT, van Dijk L, van Putten JPM, Wösten MMSM. 2017. Generation of the membrane potential and its impact on the motility, ATP production and growth in Campylobacter jejuni. Mol Microbiol 105:637–651. doi:10.1111/mmi.13723. 28586527

[79] Tremblay J, Déziel E. 2010. Gene expression in Pseudomonas aeruginosa swarming motility. BMC Genomics 11:587. doi:10.1186/1471-2164-11-587. 20961425 PMC3091734

[80] Penesyan A, Nagy SS, Kjelleberg S, Gillings MR, Paulsen IT. 2019. Rapid microevolution of biofilm cells in response to antibiotics. NPJ Biofilms Microbiomes 5:34. doi:10.1038/s41522-019-0108-3. 31728201 PMC6834608

[81] Martín-Rodríguez AJ, Rhen M, Melican K, Richter-Dahlfors A. 2020. Nitrate metabolism modulates biosynthesis of biofilm components in uropathogenic Escherichia coli and acts as a fitness factor during experimental urinary tract infection. Front Microbiol 11:26. doi:10.3389/fmicb.2020.00026. 32082279 PMC7005491

[82] Jochim A, Shi T, Belikova D, Schwarz S, Peschel A, Heilbronner S. 2019. Methionine limitation impairs pathogen expansion and biofilm formation capacity. Appl Environ Microbiol 85:e00177-19. doi:10.1128/AEM.00177-19. 30824455 PMC6495772

[83] Lazarevic V, Soldo B, Médico N, Pooley H, Bron S, Karamata D. 2005. Bacillus subtilis α-phosphoglucomutase is required for normal cell morphology and biofilm formation. Appl Environ Microbiol 71:39–45. doi:10.1128/AEM.71.1.39-45.2005. 15640167 PMC544238

