# Supplementary figures and images for: Genomic and Phenotypic Evolution of Achromobacter xylosoxidans during Chronic Airway Infections of Patients with Cystic Fibrosis
Source: mSystems. 2021 Jun 29;6(3):e00523-21. doi: 10.1128/mSystems.00523-21 (PMC8269239; doi:10.1128/mSystems.00523-21)

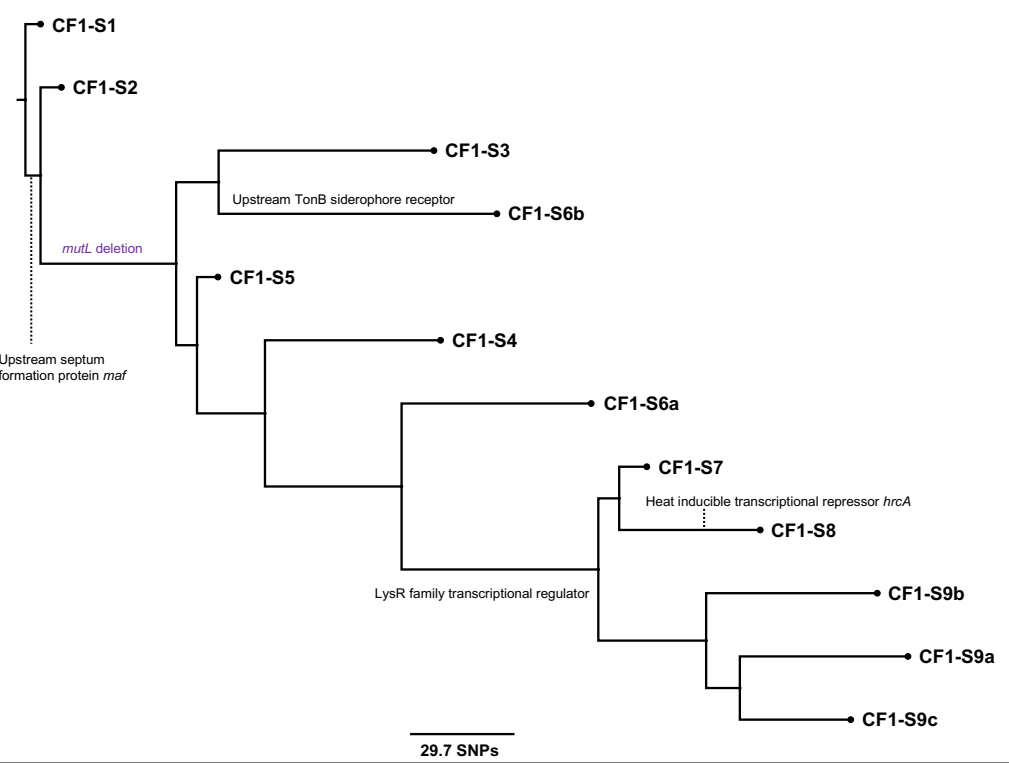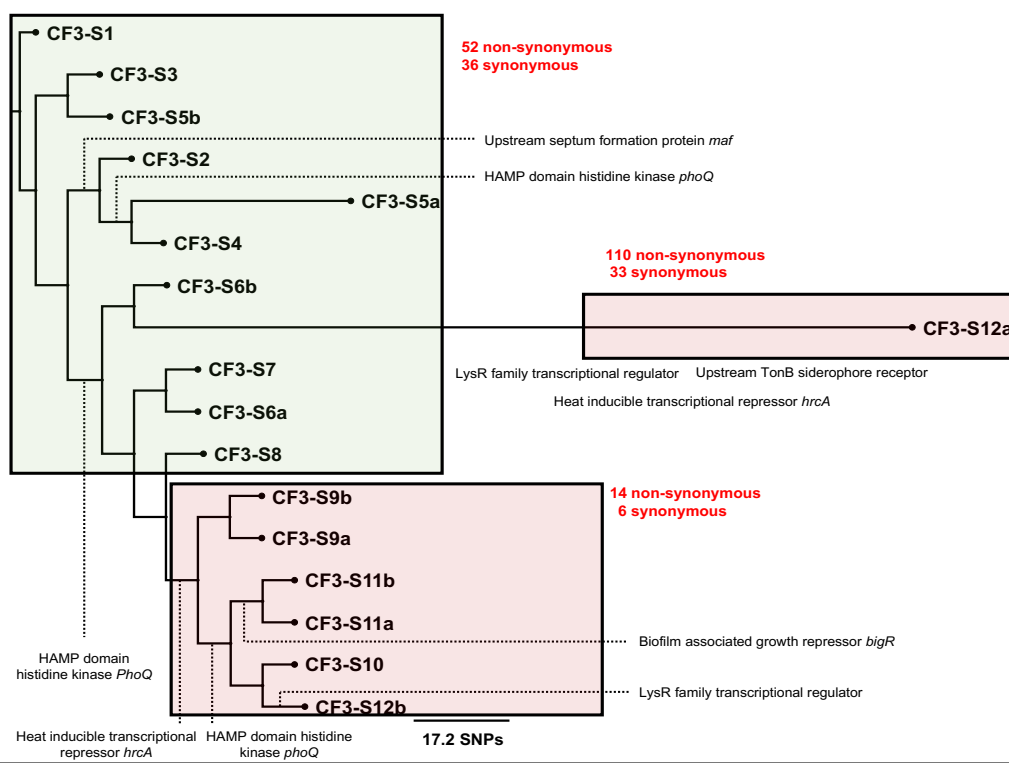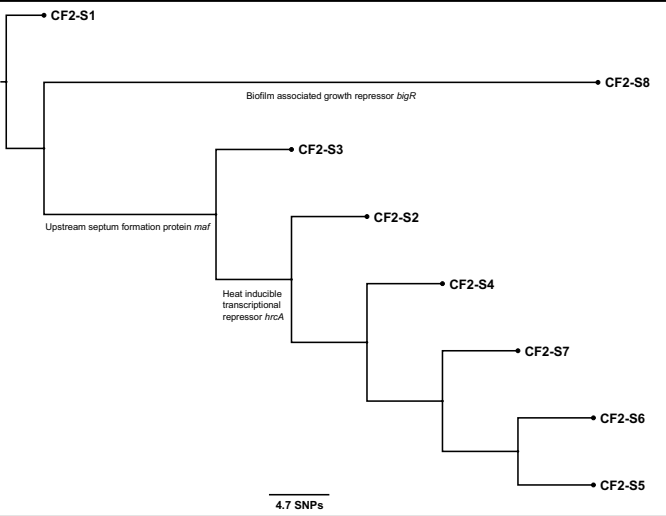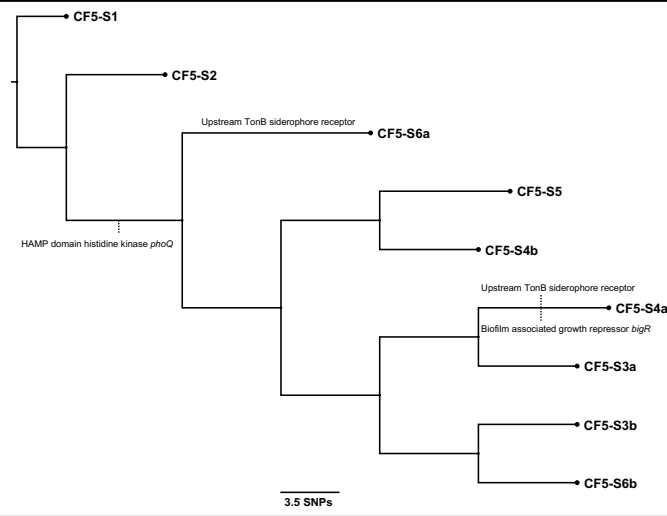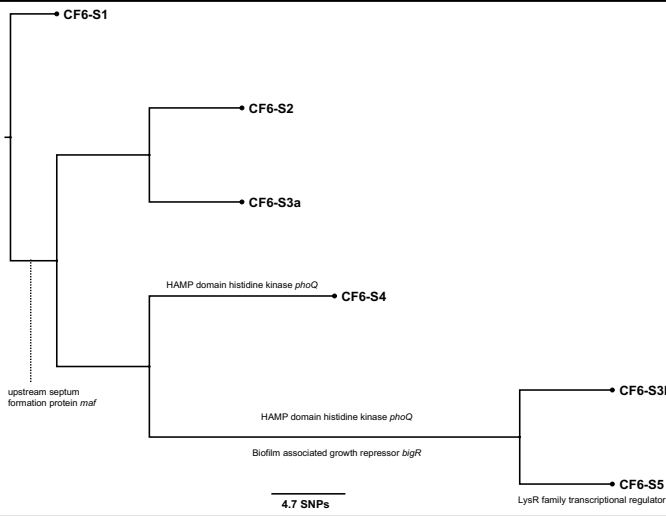

Supplement: FIG S1 [file msystems.00523-21-sf001.pdf]

# Doubling times of *A. xylosoxidans* clinical isolates in ABTGC minimal medium

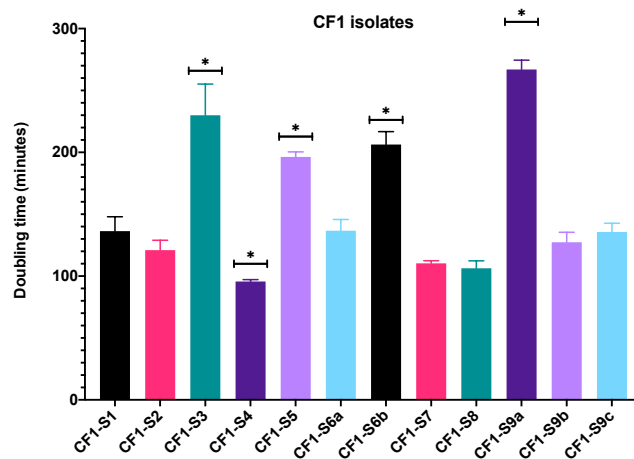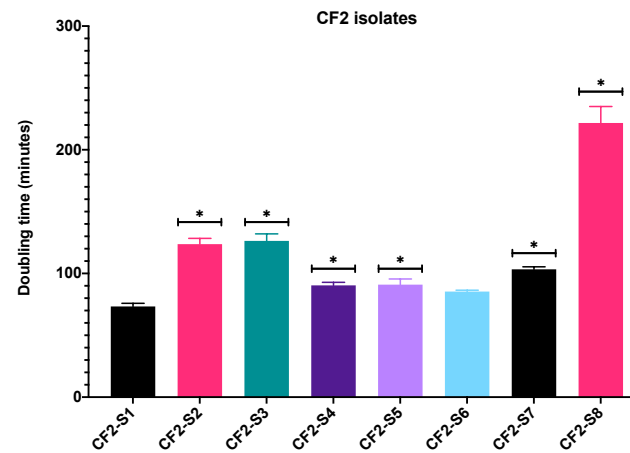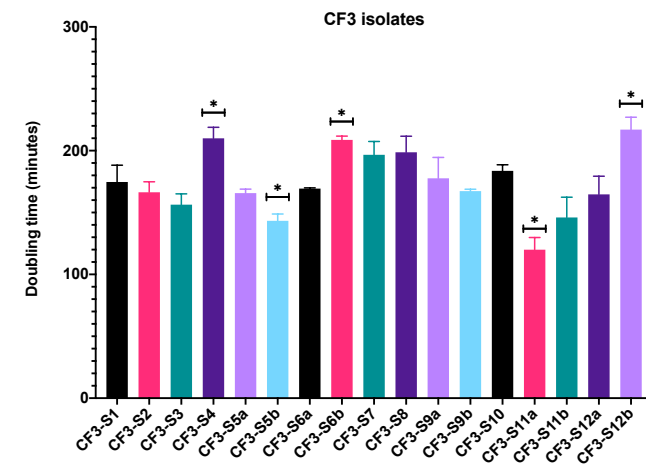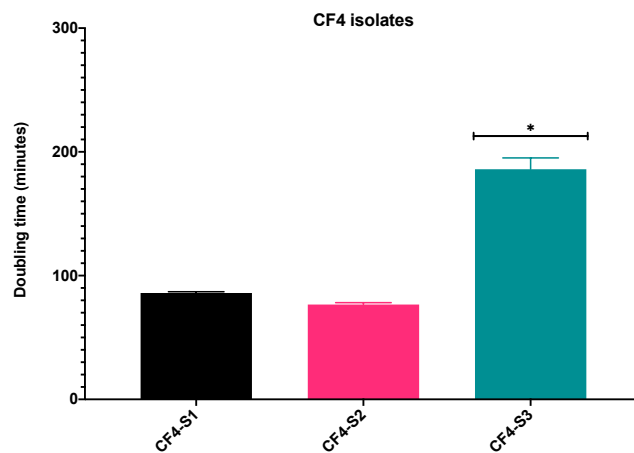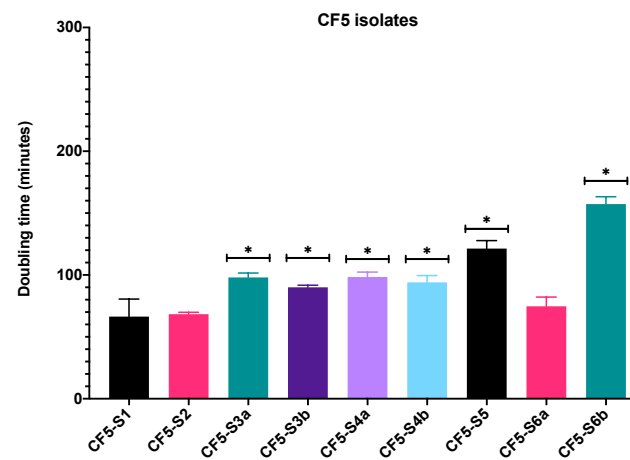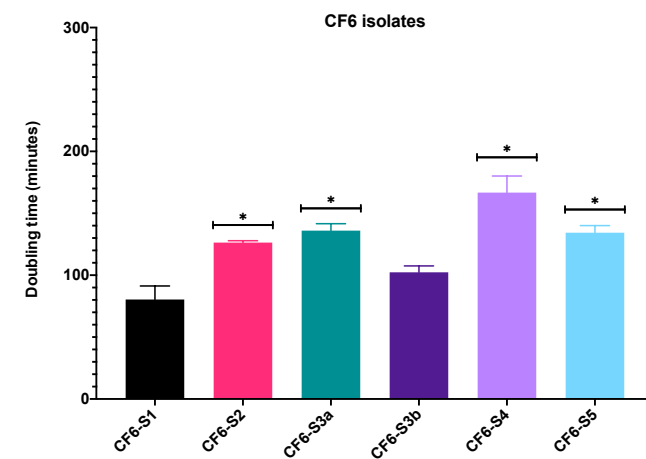

Supplement: FIG S2 [file msystems.00523-21-sf002.pdf]

# Swimming motility of *A. xylosoxidans* isolates

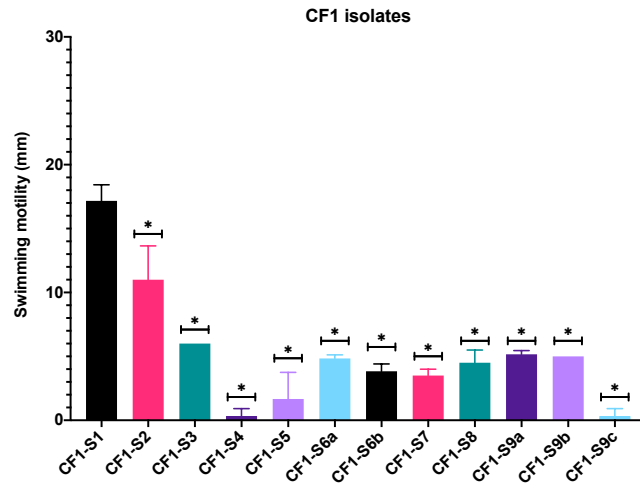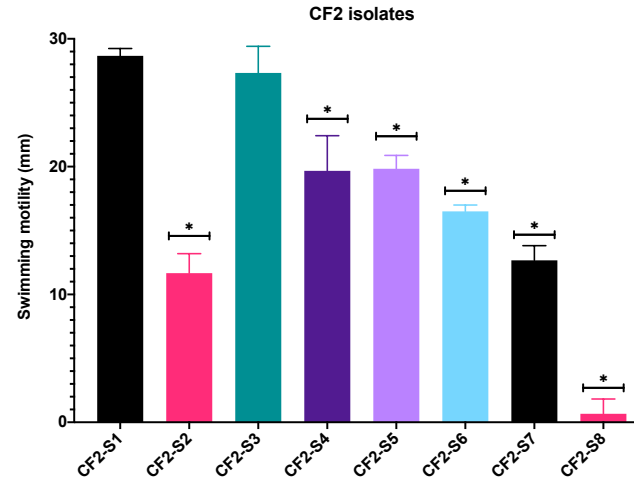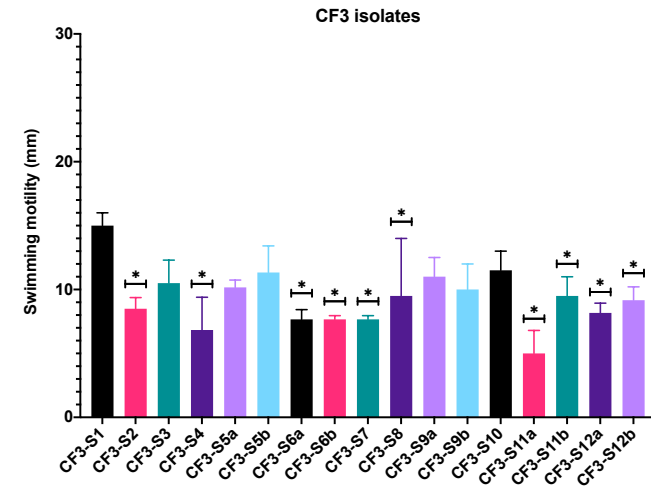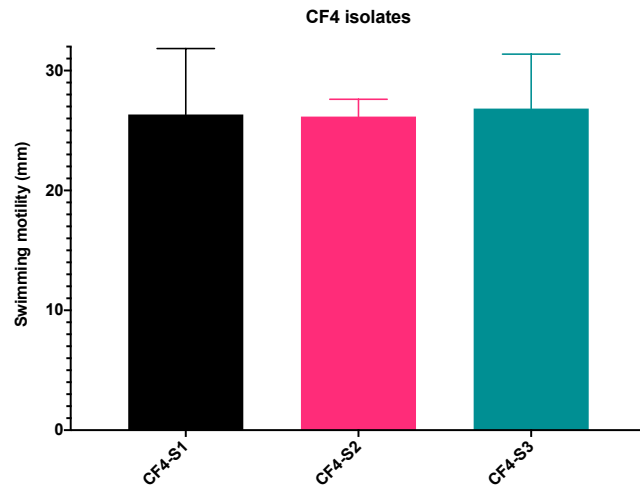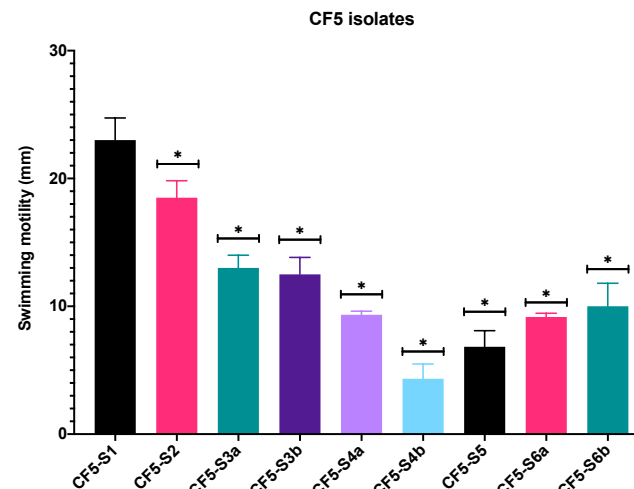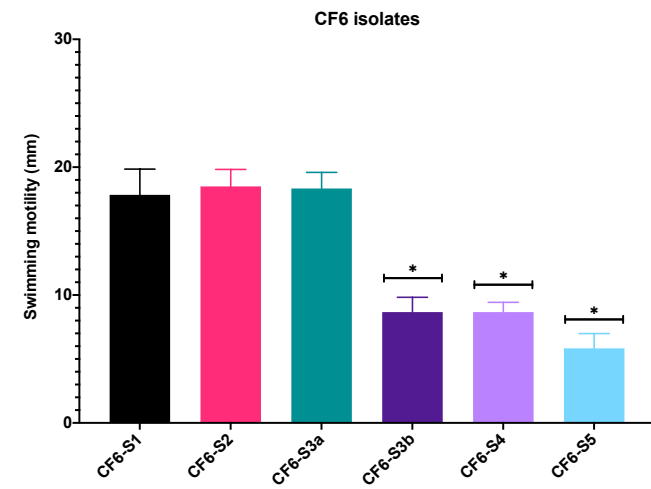

Supplement: FIG S3 [file msystems.00523-21-sf003.pdf]

# Biofilm formation of *A. xylosoxidans* clinical isolates

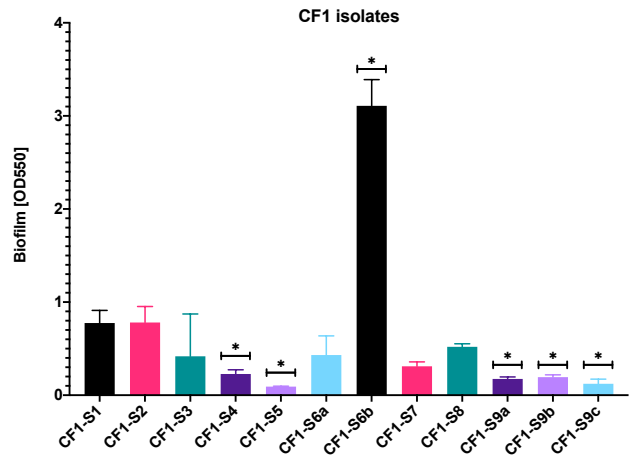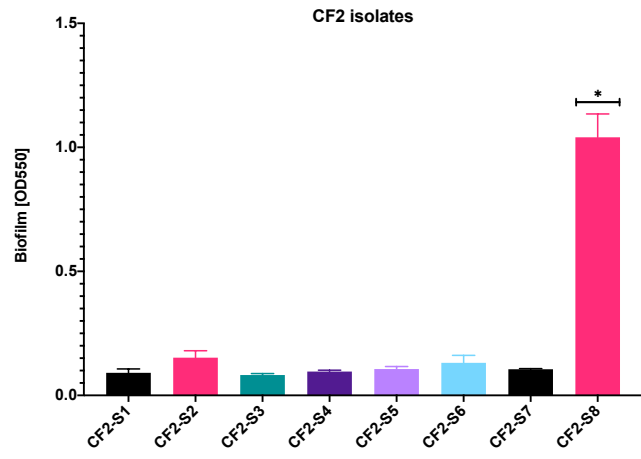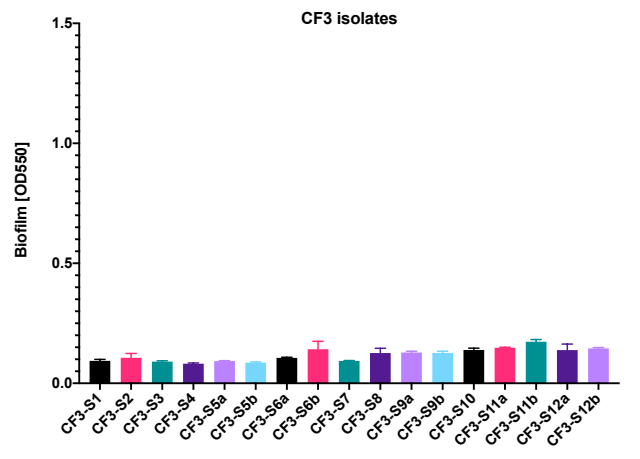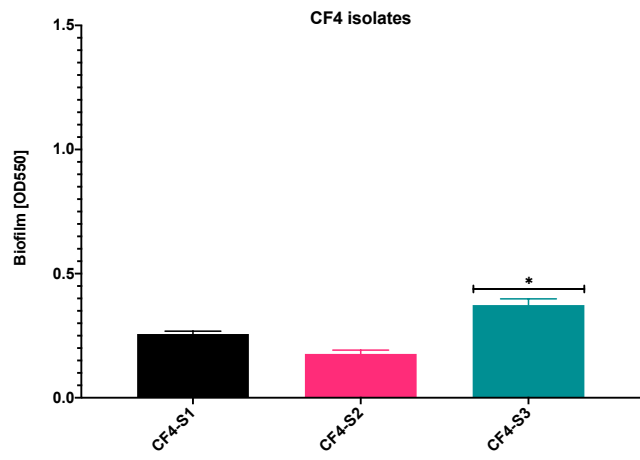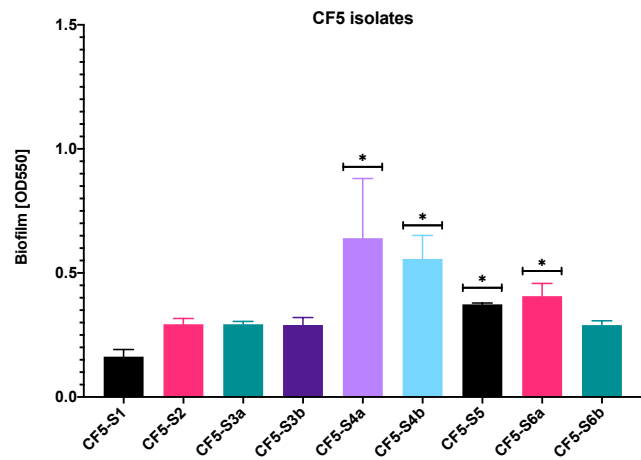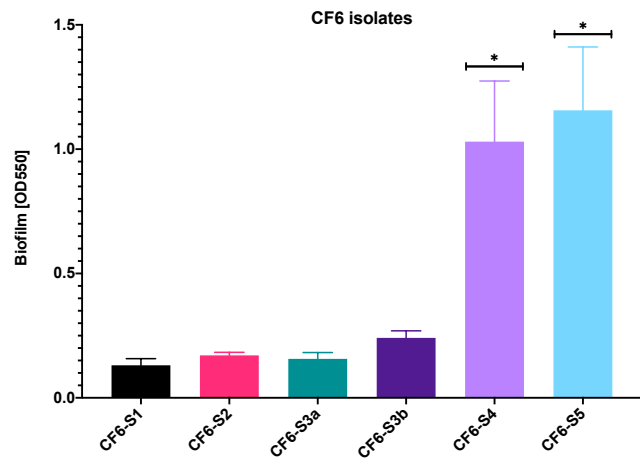

Supplement: FIG S4 [file msystems.00523-21-sf004.pdf]
